# Bootstrap Enhanced Penalized Regression for Variable Selection with Neuroimaging Data

**DOI:** 10.3389/fnins.2016.00344

**Published:** 2016-07-28

**Authors:** Samantha V. Abram, Nathaniel E. Helwig, Craig A. Moodie, Colin G. DeYoung, Angus W. MacDonald, Niels G. Waller

**Affiliations:** ^1^Department of Psychology, University of MinnesotaMinneapolis, MN, USA; ^2^School of Statistics, University of MinnesotaMinneapolis, MN, USA; ^3^Department of Psychology, Stanford UniversityStanford, CA, USA; ^4^Department of Psychiatry, University of MinnesotaMinneapolis, MN, USA

**Keywords:** penalized regression, bootstrap, fMRI, functional connectivity, individual differences, independent component analysis

## Abstract

Recent advances in fMRI research highlight the use of multivariate methods for examining whole-brain connectivity. Complementary data-driven methods are needed for determining the subset of predictors related to individual differences. Although commonly used for this purpose, ordinary least squares (OLS) regression may not be ideal due to multi-collinearity and over-fitting issues. Penalized regression is a promising and underutilized alternative to OLS regression. In this paper, we propose a nonparametric bootstrap quantile (QNT) approach for variable selection with neuroimaging data. We use real and simulated data, as well as annotated R code, to demonstrate the benefits of our proposed method. Our results illustrate the practical potential of our proposed bootstrap QNT approach. Our real data example demonstrates how our method can be used to relate individual differences in neural network connectivity with an externalizing personality measure. Also, our simulation results reveal that the QNT method is effective under a variety of data conditions. Penalized regression yields more stable estimates and sparser models than OLS regression in situations with large numbers of highly correlated neural predictors. Our results demonstrate that penalized regression is a promising method for examining associations between neural predictors and clinically relevant traits or behaviors. These findings have important implications for the growing field of functional connectivity research, where multivariate methods produce numerous, highly correlated brain networks.

## 1. Introduction

Multivariate methods for analyzing functional magnetic resonance imaging (fMRI) data, such as independent component analysis (ICA; McKeown et al., 1998), are becoming increasingly popular for exploring the brain's resting functional connectivity (Miller and D'Esposito, 2005; De Luca et al., 2006; Churchill et al., 2014). Data-driven methods like ICA are able to reduce the dimensionality of fMRI data from an immense number of voxels to a manageable number of components (or networks) with interpretable functions (Laird et al., 2011; Duff et al., 2012). However, the ICA process itself cannot tell us which networks are important to one or more criteria of interest. Thus, additional methods are needed to identify the subset of substantively important networks. This latter point is particularly relevant to clinical neuroscience research, where there is an increasing interest in understanding how the aberrant organization or functionality of large-scale brain networks contributes to psychiatric and neurological disorders (Menon, 2011). Regression models, such as ordinary least squares (OLS) regression, have often been used for this purpose (Stevens et al., 2007; Kim et al., 2009; Mennes et al., 2010; Eaton et al., 2012; Choi et al., 2013).

We advocate using penalized regression, often called regularized regression (see Zou and Hastie, 2005; Kyung et al., 2010), as a complementary data-driven method for selecting networks or neural signals related to a criterion of interest. Our specific example illustrates how to relate ICA-derived neural networks with individual differences in maladaptive behaviors; however, we will describe several additional applications for which penalized regression is highly valuable in clinical neuroscience research. In the present context, penalization refers to the shrinkage of regression coefficients toward zero (James et al., 2013, p. 204). It has been shown that this coefficient attenuation reduces model over-fitting, and when coefficients are shrunk to zero, penalized regression performs variable selection (Tibshirani, 1996, 2011; Zou and Hastie, 2005). Currently, the application of penalized regression in neuroscience research is hampered by the lack of available methods for testing the significance of penalized regression coefficients (see Lockhart et al., 2014, for a discussion).

## 2. Application of penalized regression to fMRI

We are not the first to apply penalized regression to neuroimaging data (e.g., see Valdés-Sosa et al., 2005; Carroll et al., 2009; Bunea et al., 2011; Ryali et al., 2012; Luo et al., 2013; Churchill et al., 2014; Watanabe et al., 2014; Chiang et al., 2015; Kauttonen et al., 2015). However, few studies have used penalized regression for purposes similar to ours (e.g., selecting clinical or neuroimaging biomarkers that predict behavior). One recent and promising approach for variable selection is the bootstrap enhanced elastic net proposed by Bunea et al. (2011). This method combines the nonparametric bootstrap with penalized regression to perform variable selection for neuroimaging data. Specifically, Bunea and colleagues recommend using the variable inclusion probability (VIP), which is the percentage of bootstrap replications in which a coefficient is estimated as non-zero. With a proper Bayesian interpretation (see Bunea et al., 2011), the VIP can be interpreted as the posterior probability of including the *j*-th predictor in the model. After picking an appropriate threshold, the VIP can be used to select predictors for use in follow-up analyses. Bunea et al. (2011) used a 50% threshold, but cautioned that “the threshold of 50% is user specified” (p. 1523).

Although the VIP is theoretically appealing, there has been little work exploring how the VIP performs in practice when analyzing a collection of correlated predictors that are typical of those encountered in neuroimaging research. With real neuroimaging data, determining an appropriate threshold for the VIP may be a challenging task. Bunea et al. (2011) recommend the conservative threshold of 50% because their goal is “not to miss any possibly relevant predictors” (p. 1523). It appears that this 50% threshold may view Type II Errors (False Negatives) as more costly than Type I Errors (False Positives). Uninformed use of this 50% threshold could potentially result in many False Positive findings. In neuroimaging research, attempts to replicate False Positive findings can result in substantial wastes of research resources (time and money), so a more stringent threshold may be preferred. Nonetheless, further research is needed to understand how to best use bootstrap enhanced results (with or without VIPs) to perform variable selection and parameter estimation when applying penalized regression to neuroimaging data.

## 3. Methodological objectives

In this paper, we show how computer-intensive methods, such as bootstrapping (Efron, 1979; Efron and Tibshirani, 1993) and Monte Carlo simulations, can be used to assess the significance of penalized regression coefficients in neuroimaging applications. To illustrate our proposed method, we use real neuroimaging data to predict individual differences on a self-report measure of externalizing personality traits (Krueger et al., 2002). Our procedures advance foundational work by Bunea et al. (2011) in three important ways. First, we demonstrate the relative strengths of penalized regression over OLS regression in neuroimaging datasets with highly correlated predictors. Second, we show how a novel nonparametric bootstrap quantile (QNT) confidence interval approach can be effectively used for variable selection in penalized regression models. Third, we provide a series of simulations to illustrate the validity and reliability of our proposed methods across different data conditions (i.e., different sample sizes and signal-to-noise ratios). Moreover, our study is the first (to our knowledge) to examine individual differences in ICA-derived neural connectivity metrics using penalized regression.

We have two overarching goals in this work: (i) illustrate the relative advantages of penalized regression (ridge, elastic net, and the lasso) over OLS regression when analyzing fMRI data, and (ii) compare the variable selection performance of our proposed bootstrap QNT confidence interval approach to that of the VIP. The remainder of this paper is organized as follows. Section 4 provides background on OLS and penalized regression, and also presents our proposed bootstrap QNT approach for variable selection. Section 5 presents an application to real fMRI data where the goal is to predict individual differences in externalizing traits (e.g., impulsivity, substance use) from ICA-derived connectivity networks. Section 6 illustrates how Monte Carlo simulations can be used to compare the effectiveness of different penalized regression approaches across a variety of data conditions. Section 7 presents our conclusions and general recommendations for applying penalized regression methods in functional connectivity and other neuroimaging studies. We also provide annotated R (R Core Team, 2016) code that can be used to replicate and apply our analyses, and Supplementary Material (SM) that includes additional background information on functional connectivity and externalizing.

## 4. Statistical methodology

### 4.1. OLS regression

In its simplest form, an OLS regression model has the form

(1) $$\begin{array}{l} y_{i}=\beta_{0}+\beta_{1}x_{i1}+\cdots +\beta_{p}x_{ip}+\epsilon_{i} \end{array}$$

where *y*_*i*_ denotes the criterion (dependent variable) for the *i*-th subject, *x*_*ij*_ denotes the *j*-th predictor (out of the set of *p* predictors) for the *i*-th subject, β_0_ denotes the regression intercept, β_*j*_ denotes the regression coefficient for the *j*-th predictor, and ϵ_*i*_ denotes the difference between the observed and model implied criterion for the *i*-th subject. In a typical least squares fitting procedure, the coefficients in Equation (1) are estimated by minimizing the OLS criterion:

(2) $$\begin{array}{l} F_{OLS}\left(\beta \right)=\overset{n}{\underset{i=1}{\sum }}{\left(y_{i}-\beta_{0}-\overset{p}{\underset{j=1}{\sum }}\beta_{j}x_{ij}\right)}^{2} \end{array}$$

where **β** = (β_0_, β_1_, …, β_*p*_) and *n* denotes the number of subjects.

OLS regression is a popular technique for identifying the ICA-derived networks that predict behavior (Stevens et al., 2007; Kim et al., 2009; Mennes et al., 2010; Eaton et al., 2012; Choi et al., 2013). Unfortunately, OLS regression may be ill-suited for this purpose. For instance, unless the ratio of subjects to variables is large, OLS regression will generate models that over-fit the data, such that the estimated regression coefficients may not generalize to other datasets. In addition, such models will have artificially inflated *R*^2^-values (James et al., 2013, p. 80). This is a particular concern in neuroscience research where collecting neuroimaging data is expensive and samples are often small (Button et al., 2013). In regression analyses, over-fitting may occur whenever the number of predictors approaches the number of subjects. Moreover, regression models with large numbers of predictors are difficult to interpret. Recognizing these points, researchers often prefer parsimonious models that only retain variables with substantial effects (Tibshirani, 1996).

To identify these important predictors, some researchers test multiple smaller models. However, this practice is methodologically unsound as it increases the chances of committing Type I Errors, i.e., retaining coefficients that are incorrectly deemed significantly different from zero (Simmons et al., 2011). Moreover, in OLS regression, the *p*-values that flag predictor significance are always conditional statistics in that they depend on the current predictor set. Thus, the *p*-value for a given predictor may change substantially after the inclusion or exclusion of other predictors to an existing model. This issue is particularly salient for models with highly correlated predictors (Pedhazur, 1997). Related to this problem is the associated problem of “bouncing betas” where OLS regression coefficients are unstable when computed from highly correlated variables (Darlington, 1968). This is likely to occur with fMRI data, because high correlations within and between brain networks are often present at rest and during task (Fox et al., 2005; Sporns, 2013).

### 4.2. Penalized regression

We now briefly review the mechanics of penalized regression and focus on three members of this statistical family: ridge regression (Hoerl and Kennard, 1970), the lasso (Tibshirani, 1996), and the elastic net (Zou and Hastie, 2005). The three penalized regression models that we describe in this paper can be viewed as extensions of OLS regression. As described in this section, these and other penalized regression models can often overcome the aforementioned limitations of OLS. Specifically, by adding a penalty to the OLS fit function in Equation (2), these methods can: (i) decrease coefficient estimation error and (ii) produce parsimonious and interpretable models from large numbers of highly correlated predictors (Tibshirani, 1996; Zou and Hastie, 2005). As such, penalized regression techniques represent promising alternatives to OLS methods for analyzing high dimensional data, including those used in functional connectivity studies.

Ridge regression (Hoerl and Kennard, 1970) is an early example of penalized regression that was developed to overcome known limitations of OLS regression with highly correlated predictors (Hawkins, 1975). In ridge regression, model coefficients are estimated by adding a penalty function to the OLS fit function. Specifically, ridge regression adds a quadratic shrinkage penalty to the OLS criterion to control the size of the regression coefficients. More formally, the ridge regression discrepancy function can be written

(3) $$\begin{array}{l} F_{ridge}\left(\beta \right)=F_{OLS}\left(\beta \right)+\lambda \overset{p}{\underset{j=1}{\sum }}\beta_{j}^{2} \end{array}$$

where *F*_*OLS*_(**β**) is defined in Equation (2), λ denotes a tuning parameter that ranges from 0 to infinity, and $\overset{p}{\underset{j=1}{\sum }}\beta_{j}^{2}$ denotes the quadratic shrinkage penalty, which is equal to the sum of the squared regression coefficients. The expression in Equation (3) shows that as λ approaches infinity, all coefficients are shrunk toward zero (see James et al., 2013, p. 215). However, the shrinkage rate is not equal across coefficients. Namely, larger coefficients are shrunk more than smaller coefficients (reflecting a quadratic penalty). Moreover, though not immediately apparent in Equation (3), it can be demonstrated that in ridge regression, the underlying principal components with smaller variances are shrunk closer to zero than those with higher variances (see Hastie et al., 2009, p. 67). These small-variance directions are believed to contribute most to mean-squared error, and the goal of ridge regression is to bias the solution coefficient vector away from directions that have small spread (see Frank and Friedman, 1993, p. 113). As such, ridge regression estimates are putatively more stable than their associated OLS counterparts. However, in general, ridge regression models are often not parsimonious because the method rarely attenuates coefficients to zero.

The lasso (i.e., the least-absolute-shrinkage-and-selection-operator Tibshirani, 1996) is a penalized regression approach that overcomes the aforementioned limitation of ridge regression by allowing regression coefficients to be shrunk to zero. Thus, in addition to generating robust regression coefficients with attractive out of sample properties (i.e., lasso models hold up well under cross validation), the lasso can be used as a variable selection routine when searching for parsimonious models (see James et al., 2013, p. 219). Lasso coefficients are estimated by minimizing the OLS criterion plus a penalty function that equals the sum of the absolute values of the regression coefficients. More formally, the lasso discrepancy function can be written as

(4) $$\begin{array}{l} F_{lasso}\left(\beta \right)=F_{OLS}\left(\beta \right)+\lambda \overset{p}{\underset{j=1}{\sum }}|\beta_{j}| \end{array}$$

where $\overset{p}{\underset{j=1}{\sum }}|\beta_{j}|$ denotes the sum of the absolute values of the regression coefficients, and all other terms in Equation (4) are as previously defined. In contrast to ridge regression, the lasso shrinks all coefficients by a constant amount (Tibshirani, 1996). Thus, large coefficients are retained in a model whereas smaller coefficients are shrunk to zero and thus removed from the model. Note that although each predictor in a lasso has the possibility of being included in the final model, only *n* predictors (where *n* denotes sample size) can be assigned a non-zero coefficient. Consequently, in contrast to OLS regression, a lasso regression can be estimated in data sets that contain more predictors than observations. This feature has made the lasso a popular tool in GWAS research (D'Angelo et al., 2009; Brown et al., 2011; Ayers and Cordell, 2013) where the number of predictors can range in the thousands. Later, we show how the lasso can also be profitably used in data sets where *n* > *p*, and can produce better results than OLS when *n* ≈ *p*.

The third penalized regression algorithm that we describe in this paper is called the elastic net (Zou and Hastie, 2005). As shown below, this model can be conceptualized as a combination of ridge regression with the lasso. More formally, the elastic net includes a composite penalty function that equals a weighted sum of the ridge and lasso penalty functions (Friedman et al., 2010). This composite penalty putatively allows the elastic net to enjoy the methodological advantages of both ridge regression and the lasso (Cho et al., 2010). The elastic net discrepancy function has the form

(5) $$\begin{array}{l} F_{enet}\left(\beta \right)=F_{OLS}\left(\beta \right)+\lambda P_{\alpha }\left(\beta \right) \end{array}$$

where

(6) $$\begin{array}{l} P_{\alpha }\left(\beta \right)=\frac{1}{2}\left(1-\alpha \right)\overset{p}{\underset{j=1}{\sum }}\beta_{j}^{2}+\alpha \overset{p}{\underset{j=1}{\sum }}|\beta_{j}|. \end{array}$$

denotes the elastic net penalty (Zou and Hastie, 2005). Notice in Equation (6) that, as mentioned earlier, the elastic net penalty is a composite function that is composed of two component penalty functions. The first penalty minimizes the weighted sum of squared regression coefficients and thus equals the ridge penalty, whereas the second component minimizes the weighted sum of absolute regression coefficients and thus equals the lasso penalty. The relative contribution of the two component penalties is controlled by a tuning parameter, α, that ranges from 0 to 1. In effect, α controls the proportional contribution of the ridge regression and lasso penalties. For example, when λ > 0 and α = 0, Equation (5) reduces to the ridge regression discrepancy function in Equation (3). When λ > 0 and α = 1, Equation (5) reduces to the lasso discrepancy function in Equation (4). Whenever λ > 0 and 0 < α < 1, Equation (5) defines the elastic net.

The elastic net, like the lasso, can achieve coefficient shrinkage and variable selection (Zou and Hastie, 2005). Unlike the lasso, but similar to ridge regression, the elastic net can retain more than *n* variables in data sets in which *p* > *n*. Moreover, the elastic net has a unique “grouping effect” (Zou and Hastie, 2005) property that is relevant when analyzing data sets with subsets of highly correlated variables. Whereas, the lasso in these cases will arbitrarily select one variable from a cluster of correlated variables, the elastic net assigns all variables within a correlated subset a common coefficient. Thus, when using the elastic net, highly correlated variables are retained or discarded as a set in the final model. This singular characteristic of the elastic net is particularly salient for fMRI data given the potential high collinearity between brain networks. In summary, the elastic net is a convenient method for finding sparse and interpretable regression models in data sets with large numbers of predictors and possibly smaller numbers of observations. Data sets with these characteristics are common in neuroscience research.

### 4.3. Tuning parameter and variable selection

All three penalized regression methods that were introduced in the previous sections include tuning parameters to calibrate *P*_α_(**β**). We used the glmnet package (Friedman et al., 2010) in the R programming language to implement the elastic net. This package selects the optimal value of λ using *k*-fold cross-validation (see James et al., 2013, p. 254). In *k*-fold cross-validation, a data set is randomly parsed into *k* subsets or folds. Typically, each fold contains an equal sized subset of the data. Using these subsets, *k* − 1 folds are combined into a training set and the remaining fold serves as a test (or holdout) sample. A model is fit to the training data and then applied to the holdout sample to calculate a cross-validated mean-squared error (CV-MSE). This process is repeated by systematically creating training sets that exclude a single member of the original folds. The glmnet package implements this procedure by evaluating each term in a λ sequence such that the lowest λ value generates a regression model that includes all predictors, and the highest λ value generates an intercept only regression model that excludes all predictors. Unfortunately, the glmnet package does not include a function for locating the optimal value of α. Thus, at present, researchers must produce their own code for locating optimal α values. Many researchers tune α and λ simultaneously using cross-validation (Cho et al., 2009, 2010; Li and Li, 2010; Bunea et al., 2011; Kohannim et al., 2012). Other researchers use preselected α values and then estimate λ by cross-validation (Hautamäki et al., 2011; Shen et al., 2011; Li et al., 2013). In the analyses described below, we employed the latter approach.

Once tuning parameters have been selected, a logical next step is to identify regression coefficients that are significantly different from zero. Unfortunately, there are no established methods for obtaining accurate *p*-values and confidence intervals for elastic net and lasso regression coefficients (although see Lockhart et al., 2014, for a novel method for calculating asymptotically valid *p*-values for the lasso). Fortunately, by using computer intensive methods (e.g., the bootstrap or jackknife), researchers are able to evaluate the significance of penalized regression coefficients via sample splitting (Meinshausen et al., 2009; Wasserman and Roeder, 2009) or resampling methods (D'Angelo et al., 2009; Bunea et al., 2011; Tibshirani, 2011). However, available research has not reached a consensus on how to define standard errors or confidence intervals for lasso estimates (see Tibshirani, 1996; Osborne et al., 2000; Kyung et al., 2010; Tibshirani, 2011). One source of this debate is that several techniques for calculating lasso standard errors assign standard errors of zero to excluded predictors (see Tibshirani, 1996; Osborne et al., 2000; Chatterjee and Lahiri, 2011). Nonparametric bootstrapping is advantageous in this regard, as even an excluded predictor can have a non-zero standard error if said predictor has a non-zero estimated coefficient in at least one bootstrap sample (Tibshirani, 2011).

### 4.4. Enhancing penalized regression with the bootstrap

We begin by reviewing the bootstrap enhanced (BE) procedure proposed by Bunea et al.(2011, p.1522). The underlying logic of this procedure can be summarized as follows:

1. [Model fitting] For each value of λ and α, complete the following steps:
  - [Elastic net] Fit elastic net on all standardized predictors.
  - [Bias correction] Fit OLS model using predictors selected by elastic net.
2. [Parameter tuning] Apply *k*-fold CV to find λ and/or α that minimizes CV-MSE.
3. [Bootstrap] Apply Steps 1–2 to each of *B* bootstrap samples.

The bias-correction (Step 1b) is meant to correct the bias (shrinkage) induced by the penalized regression fitting in Step 1a. It should be noted that this bias-correction step is not necessary to select tuning parameters in penalized regression models. For example, this bias-correction step is not implemented in popular software such as the glmnet package mentioned previously (Friedman et al., 2010). Moreover, due to the bias vs. variance trade-off, it is unclear whether bias-correction at this particular juncture produces better coefficient estimates from a mean-squared error perspective. Further research is therefore needed to understand the properties of bias-corrected penalized regression coefficients. Because it is unclear whether the added computation involved with the bias-correction is worthwhile, we do not implement the bias-correction step for variable selection in this paper.

Applying the BE method produces a bootstrap distribution for each regression coefficient, β_*j*_. The information in this bootstrap distribution can be used to assess the influence of the predictor β_*j*_ in the model. Colloquially, if the bootstrap distribution for β_*j*_ shows a “large” difference from zero, we can conclude that the *j*-th predictor may be important for the model. How to quantify a “large” difference from zero is one of the topics that we explore in this paper. One possible approach is Bunea et al.'s (2011) VIP, which quantifies the importance of the *j*-th predictor as the proportion of times (out of the *B* bootstrap replicates) the *j*-th predictor receives a non-zero coefficient estimate. With a Bayesian interpretation (i.e., Laplace prior on β_*j*_), the VIP can be understood as the posterior probability of including the *j*-th predictor in the model (see Bunea et al., 2011). However, for real neuroimaging data, there is no theoretical reason that this Laplace prior should be preferred. Furthermore, it should be noted that by dichotomizing (zero vs. non-zero) the estimated predictor coefficients, the VIP does not take advantage of coefficient magnitude information that could be useful for variable selection.

To incorporate magnitude information into the variable selection process, we propose using the quantiles of the bootstrap distribution of β_*j*_ to determine the significance of the *j*-th predictor. Given a significance threshold 1 − α^*^, the *j*-th predictor is selected with threshold 1 − α^*^ if the 100(1 − α^*^)% bootstrap confidence interval for β_*j*_ does not contain zero. The 100(1 − α^*^)% bootstrap confidence interval is given by $\left[Q_{j,\alpha^{*}∕2};Q_{j,1-\alpha^{*}∕2}\right]$, where *Q*_*j*, α_ denotes the quantile value such that $\alpha =\frac{1}{B}\overset{B}{\underset{b=1}{\sum }}1_{\left\{{\hat{\beta }}_{jb}\;\leq \;Q_{j,\alpha }\right\}}$, where 1_{·}_ is an indicator function that equals 1 if the argument within braces is true and equals 0 otherwise, and ${\hat{\beta }}_{jb}$ denotes the estimate of β_*j*_ in the *b*-th bootstrap replicate. Compared to the VIP, our proposed quantile approach produces a more stringent variable selection rule, as evidenced by the following theorem.

Theorem 1. *For the same significance threshold* 1 − α^*^, *the number of predictors declared significant by VIP selection will be greater than or equal to the number of predictors declared significant by quantile (QNT) selection*.

To prove Theorem 1, we need to show that (i) if β_*j*_ is selected by QNT, then β_*j*_ must also be selected by VIP, and (ii) if β_*j*_ is not selected by VIP, then β_*j*_ must not be selected by QNT. Proof of part (i) is straightforward: if β_*j*_ is selected by QNT with threshold 1 − α^*^, then (by definition) zero is not contained within $\left[Q_{j,\alpha^{*}∕2};Q_{j,1-\alpha^{*}∕2}\right]$, which implies that $VIP_{j}>1-\alpha^{*}$. To prove part (ii), we offer a simple proof by contradiction. Suppose that the VIP does not select β_*j*_ as significant (i.e., $VIP_{j}<1-\alpha^{*}$), but QNT selects β_*j*_ as significant (i.e., zero is not in $\left[Q_{j,\alpha^{*}∕2};Q_{j,1-\alpha^{*}∕2}\right]$). Clearly, this creates a contradiction because $VIP_{j}<1-\alpha^{*}$ implies that at least 100α^*^% of the coefficients are zero across bootstrap replications, which implies that zero must be within $\left[Q_{j,\alpha^{*}∕2};Q_{j,1-\alpha^{*}∕2}\right]$.

After applying the VIP and/or QNT approach to select predictors, it may be desirable to obtain final coefficient estimates corresponding to the selected predictors. Note that the penalized coefficient estimates from the full model could be considered undesirable for two reasons (i) the penalization adds bias to the solution, and (ii) these parameter estimates are conditioned on the other (insignificant) predictors in the model. One simple approach to obtain final coefficient estimates is bias correction, i.e., fit a restricted OLS model using the selected predictors. This sort of approach has been applied by Efron et al. (2004) to obtain bias-corrected coefficients after using the Least Angle Regression algorithm to select predictors. Note that Bunea et al. (2011) propose this sort of bias-correction for the tuning parameter selection, which is different from our use of the bias-correction. In contrast, we propose the use of bias-correction to obtain final coefficient estimates corresponding to the predictors that are determined relevant for the model.

## 5. Application to real fMRI data

### 5.1. Data

To demonstrate the utility of penalized regression for functional connectivity analyses, we used a moderately large neuroimaging dataset (*n* = 122) with *p* = 27 spatial components determined via ICA^1^. Specifically, we used a subject-level metric derived from these components (see Section 5.2 for details on the metric computation). Although the ICA-generated components were spatially uncorrelated at the group level, the 27 subject-level metrics calculated based on these components were highly correlated across subjects, with correlations ranging from 0.14 to 0.77. The criterion for our regression analyses was the Externalizing Spectrum Inventory (Krueger et al., 2007), which is a composite of externalizing traits and behaviors (e.g., impulsivity and prior thefts) that have been identified as important markers of psychopathology (Krueger et al., 2002). See the Supplementary Material for background information on the neural basis of externalizing.

The *n* = 122 male participants were recruited from the community via the CraigsList.org website, and were between the ages of 20 and 40 (mean age = 25.7 years). The University of Minnesota institutional review board approved the study and participants provided written informed consent. Subjects were excluded for neurological and psychiatric disorders, regular psychotropic drug use, MRI contraindications (e.g., ferromagnetic implants, non-removable piercings, pacemakers), and excessive movement (mean absolute displacement above 1.5 mm, or any absolute displacement above 2.75 mm). Neuroimaging data were acquired on a 3T Siemens scanner at the University of Minnesota's Center for Magnetic Resonance Research, and resting state scans were 5 min long^2^. During the scan, researchers assured that participants remained awake by having them push a button each time a fixation cross changed from gray to white or vice versa (occurred 5 times). High-resolution T1-weighted structural scans were collected for registration.

**Figure 1:**
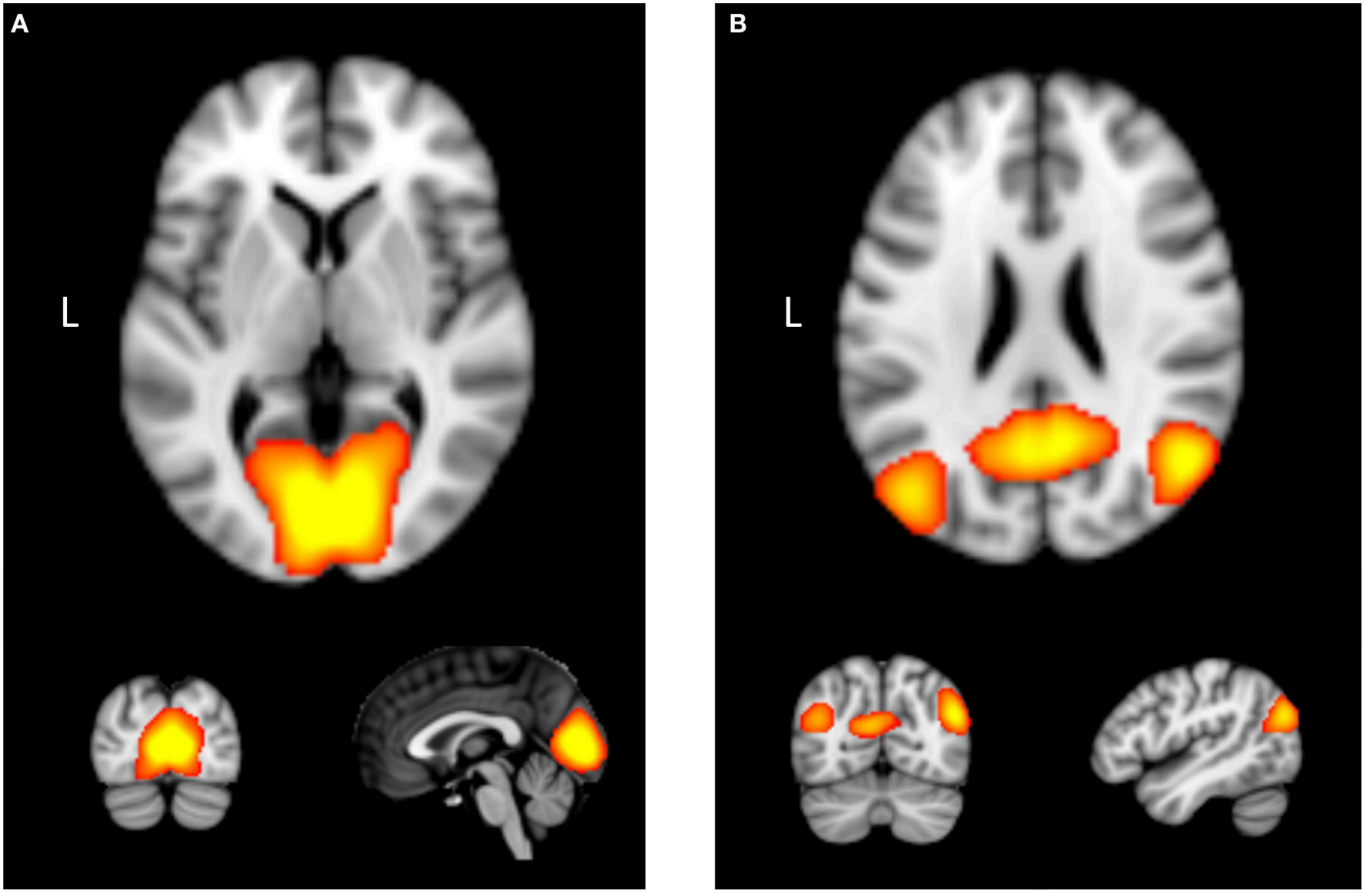
**Examples of two intrinsic connectivity networks derived using independent component analysis with functional neuroimaging data: (A)** medial vision network, which contains occipital cortex. **(B)** Posterior portion of the “default mode network,” which contains areas such as the posterior cingulate cortex, precuneus, and bilateral angular gyrus.

Prior to performing the penalized regression, we processed the fMRI data using the following procedures. Data were pre-processed using FSL's MELODIC Toolkit^3^. Pre-processing procedures included registration to T1-weighted structural image, brain extraction, grand mean intensity normalization of the 4D dataset, high pass temporal filtering, motion correction, and motion regression as the final step. Resting state data were then decomposed using the spatial ICA algorithm in the MELODIC toolkit, which assumes spatial independence between components (Calhoun et al., 2001). In the Supplementary Material we detail our specific procedures for employing ICA with resting fMRI data. Artifacts were visually identified following procedures outlined by Kelly et al. (2010); this step resulted in 27 non-artifactual ICNs. Non-artifactual ICNs covered a range of neural functions, which included: vision, audition, motor, and visuospatial processing as identified by Laird et al. (2011), see Figure 1.

### 5.2. Analyses

Dual-regression was used to derive subject specific maps and time series for each individual based on the group-level maps derived from the ICA^4^ (Beckmann et al., 2009; Zuo et al., 2010; Poppe et al., 2013; Wisner et al., 2013a; Moodie et al., 2014). First, the complete set of group-level spatial maps was used as spatial regressors for each subject's 4D dataset. This process yielded a set of subject-specific time series, with one per group-level spatial map for each subject. We elected to apply the group-level spatial maps derived from the present sample in the dual regression, given our relatively large sample size when compared with other publicly available maps. Second, subject-specific time series were used as temporal regressors for the respective subjects' 4D dataset to derive a set of subject-specific spatial maps. The value of each voxel in a particular subject's spatial map reflected how well the time series of that voxel corresponded to the overall time series of the component, for the respective subject.

**Figure 2:**
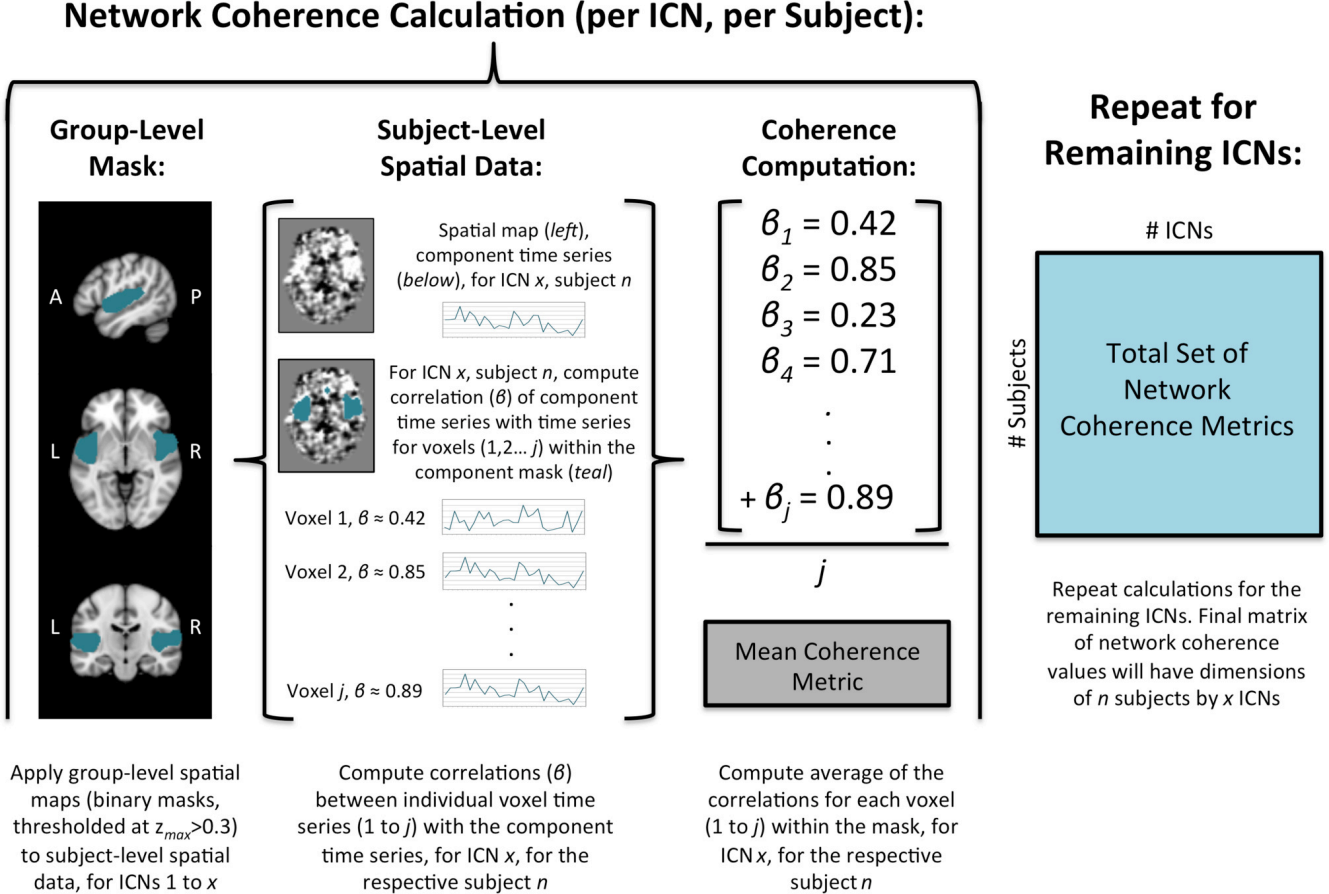
**Illustration of network coherence calculation using ICA with dual-regression**.

Network coherence (within-network connectivity) was calculated for each subject (for all ICNs) using the subject-level spatial maps from the dual-regression procedure (see Figure 2). First, group-level components were normalized by the maximum value and then thresholded at values of zmax > 0.30 (Poppe et al., 2013). Second, the thresholded group-level component maps were binarized and subsequently applied as masks to the respective subject-level spatial maps. Third, the average of the coherence values for all voxels within the respective group-level mask for each component was computed (Wisner et al., 2013b). This process was repeated for all subjects to yield average coherence values for the 27 non-artifactual ICNs for all subjects. For each subject, these values represent the overall voxel coherence within each network; larger values reflect greater coherence over time. Specifically, these metrics represent ICA network coherence, which we distinguish from the spectral coherence described in the signal processing literature.

The purpose of this example is to compare variable selection results using OLS regression vs. BE penalized regression techniques when analyzing real neuroimaging data. We compared five different versions of the elastic net by fixing α at five values α ∈ {0, 0.25, 0.5, 0.75, 1}. For each of the five elastic net models, we used the cv.glmnet function (Friedman et al., 2010) to perform 5-fold cross-validation to select the optimal λ. To make the OLS results comparable to the penalized regression results, we applied the bootstrap procedure to the OLS results as well. This means that for each of the *B* = 5000 bootstrap samples, we applied six different methods: OLS and five elastic net models. We compared the variable selection results using both the VIP and QNT methods with various significance thresholds: 1 − α^*^ ∈ {0.5, 0.55, …, 0.9, 0.95}. We refer readers to the R code in the SOM for further details on our analysis procedure.

### 5.3. Results

**Figure 3:**
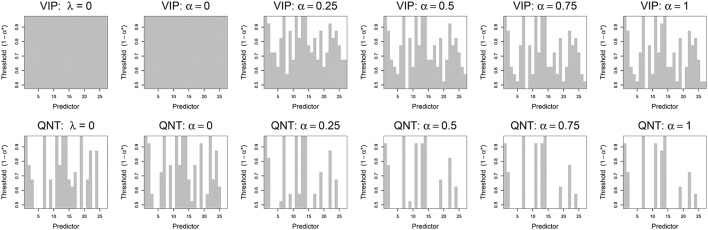
**Results from real data analysis using the VIP approach (top) and the QNT approach (bottom)**. Each column represents a different regression model: OLS (λ = 0), ridge regression (α = 0), elastic net (α = 0.25, 0.5, 0.75), and the lasso (α = 1). Vertical gray bars indicate whether a specific predictor, i.e., network derived using ICA, was selected at a given significance threshold.

In Figure 3 we plot the variable selection results for the real data using both the VIP and QNT approaches. For each subplot of Figure 3, a gray box is plotted if β_*j*_ (abscissa) is declared significant using the corresponding significance threshold (ordinate), where the threshold is 1 − α^*^ for both variable selection methods. Note that β_*j*_ is declared significant if $VIP_{j}>1-\alpha^{*}$ (for VIP selection) or if $0\notin \left[Q_{j,\alpha^{*}∕2};Q_{j,1-\alpha^{*}∕2}\right]$ (for QNT selection). As a first point, note that the VIP is not useful for the OLS (λ = 0) and ridge (α = 0) solutions, given that OLS and ridge will not typically zero-out any coefficients. In contrast, the QNT approach can be meaningfully applied to the OLS and ridge results, as well as the other (non-ridge) elastic net results. As a second point, note that the QNT approach always produced a sparser solution than the VIP approach for the same significance level, which is expected given the implications of Theorem 1.

When we applied the VIP with the conservative 50% threshold, the various models selected anywhere from 25 to 27 predictors as significant (out of 27 total predictors). The exact number of predictors selected varied slightly as a function of the elastic net, α tuning parameter. At more stringent thresholds (e.g., 70–90%), the VIP still selected upwards of 15 predictors as significant. The most parsimonious VIP-suggested model (at a 95% threshold) contained four predictors: ICN1 (medial vision), ICN7 (posterior insula and Heschl's Gyrus), ICN11 (anterior insula, ventral striatum, and anterior cingulate cortex), and ICN14 (anterior insula, and orbitofrontal cortex). In contrast, the most parsimonious QNT-suggested model (at a 95% threshold) contained only ICN7 and ICN14.

In Table 1 we display the coefficient estimates for the fitted regression models. Note that OLS-All refers to the non-BE OLS solution, where coefficient significance was determined using normal theory asymptotic results. Also, notice that the three sparsest BE penalized regression models (α = 0.50, 0.75, 1) have identical coefficient estimates, thus we present the results for these models in a single column of the table. For these three α levels, the same two predictors (ICNs 7 and 14) were included in the final OLS model that we used to obtain bias-corrected estimates. Table 1 also contains information regarding model strength. In particular, our findings indicate that the more parsimonious models had smaller *R*^2^-values. Consequently, compared to the sparser penalized solutions, the OLS solution was overly-optimistic about the model's ability to explain variation in externalizing scores. This effect is more dramatic if we consider the full OLS model with 27 predictors, which produced an *R*^2^ of 0.46 and an Adjusted-*R*^2^ of 0.31. Interestingly, we also found that the CV-MSE was almost identical for the 5 elastic net variations (see Figure S1). We investigated these model fit issues (and other issues) in the following simulation study.

**Table 1:** **Coefficient estimates for OLS, ridge regression, elastic net, and the lasso**.

**Predictor**	**OLS-All**	**OLS (λ = 0)**	**Ridge (α = 0)**	**E-Net (α = 0.25)**	**E-Net (α ≥ 0.5)**
ICN1: Medial Vision	−**0.47**	−**0.16**	−**0.26**	−**0.14**	0.00
ICN2: R Fronto-Parietal	−0.19	0.00	0.00	0.00	0.00
ICN3: Bilateral Supramarginal Gyrus	−0.15	0.00	0.00	0.00	0.00
ICN4: L Postcentral Gyrus	0.05	0.00	0.00	0.00	0.00
ICN5: Bilateral IFG	0.12	0.00	0.00	0.00	0.00
ICN6: Lateral Occipital + Precuneus + PCC	0.09	0.00	0.00	0.00	0.00
ICN7: Insula + Heschl's Gyrus	**0.39**	**0.37**	**0.33**	**0.37**	**0.32**
ICN8: Bilateral Angular Gyrus	−0.02	0.00	0.00	0.00	0.00
ICN9: Putamen + Amygdala	−0.17	0.00	0.00	0.00	0.00
ICN10: Bilateral Superior Temporal Gyrus	−0.02	0.00	0.00	0.00	0.00
ICN11: Insula + Ventral Striatum + ACC	−**0.44**	−**0.38**	−**0.35**	−**0.29**	0.00
ICN12: Posterior Vision	0.18	0.00	0.00	0.00	0.00
ICN13: Parietal/Occipital Cortices	**0.32**	**0.41**	**0.37**	**0.41**	0.00
ICN14: Bilateral Insula and OFC	−**0.49**	−**0.45**	−**0.30**	−**0.29**	−**0.38**
ICN15: Bilateral Frontal Pole	−0.17	0.00	0.00	0.00	0.00
ICN16: Supplementary Motor	0.15	0.00	0.00	0.00	0.00
ICN17: Primary Motor	0.19	0.00	0.00	0.00	0.00
ICN18: Motor	−0.07	0.00	0.00	0.00	0.00
ICN19: Frontal Medial Cortex + ACC	**0.31**	**0.31**	0.00	0.00	0.00
ICN20: Precuneus + PCC	−0.03	0.00	0.00	0.00	0.00
ICN21: Occipital Pole	0.15	0.00	0.00	0.00	0.00
ICN22: R Postcentral Gyrus	0.26	0.00	**0.33**	0.00	0.00
ICN23: Cerebellum	0.04	0.00	0.00	0.00	0.00
ICN24: Subcallosal Cortex + OFC	0.19	0.00	0.00	0.00	0.00
ICN25: Inferior Lateral Occipital	0.07	0.00	0.00	0.00	0.00
ICN26: Precuneus	−0.09	0.00	0.00	0.00	0.00
ICN27: Hippocampus + Amygdala + TP	−0.01	0.00	0.00	0.00	0.00
*R*^2^ (Error Standard Deviation)	0.46 (0.83)	0.27 (0.88)	0.29 (0.86)	0.23 (0.90)	0.12 (0.95)

*Coefficients that are significant at a 95% threshold are indicated with boldfaced font. IFG, Inferior Frontal Gyrus; ACC, Anterior Cingulate Cortex; OFC, Orbitofrontal Cortex; PCC, Posterior Cingulate Cortex; TP, Temporal Pole*.

## 6. Application to simulated fMRI data

### 6.1. Design

We designed a simulation study to evaluate and compare the effectiveness of OLS and various penalized regression procedures when analyzing simulated neuroimaging data. To ensure that our simulation results were relevant to our problem, our simulation design was motivated by our real data results. In the real data example, we found that only ICNs 7 and 14 were selected as significant using the lasso with a stringent QNT significance threshold of 95%. Consequently, we designed a simulation study where ICN 7 and 14 were the only active predictors, and the other 25 predictors were inactive (i.e., had coefficients of zero). The purpose of the simulation was to determine how often each predictor (of the 27 predictors) was “selected as significant” using a variety of sample sizes (*n*), error standard deviations (σ), and selection methods (e.g., VIP vs. QNT).

In the simulation, the two active coefficients were set at β_7_ = 0.3 and β_14_ = −0.4; these values were inspired by the least-squares estimates from the OLS model that included only ICNs 7 and 14 as predictors: ${\hat{\beta }}_{7}=0.32$ and ${\hat{\beta }}_{14}=-0.38$ (see Table 1). We manipulated two simulation conditions: (i) sample size (4 levels; *n* ∈ {30, 60, 90, 122}), and (ii) error standard deviation (3 levels; σ ∈ {0.5, 0.7, 1}). Treating the *n* = 122 subjects as the population, the three σ values correspond to *R*^2^ ∈ {0.33, 0.2, 0.11}, respectively. These *n* and σ values were selected to cover the range of values encountered in typical neuroimaging studies. In our real data example with only ICNs 7 and 14 included in the model, the estimated error standard deviation is about $\hat{\sigma }=0.95$.

Our data generation procedure was as follows. To facilitate comparisons between the simulation and the real data results, we used the design matrix **X**, from the real data to generate simulated data. For each sample size *n*, we randomly sampled (without replacement) *n* subjects from our sample of 122 observed response vectors, and used the corresponding *n* rows of **X** as the true predictors. The true response variable was defined as

$$y_{i}=0.3x_{i7}-0.4x_{i14}+\epsilon_{i}$$

where $\epsilon_{i}\overset{iid}{\sim }N\left(0,\sigma^{2}\right)$ is independently, randomly sampled from a normal distribution with mean zero and variance σ^2^. We repeated this data-generation process 100 times for each of the 12 (4 *n* × 3 σ) cells of the simulation design.

### 6.2. Analyses

For each simulation replication (or generated dataset), we compared six different regression methods: (i) OLS regression, (ii) ridge regression, (iii)–(v) elastic net with α = 0.25, 0.5, 0.75, and (vi) lasso. For each method, we used *B* = 5000 bootstrap samples to determine the bootstrap distribution of each of the 27 coefficients. Given the bootstrap distributions, we used the VIP (Bunea et al., 2011) and the QNT to determine the significance of each predictor at ten thresholds of interest, i.e., 1 − α^*^ ∈ {0.5, 0.55, …, 0.9, 0.95}. Note that the VIP approach can only be meaningfully applied to methods (iii) through (vi), whereas the QNT approach is applicable to all methods.

### 6.3. Results

#### 6.3.1. Overview

**Figure 4:**
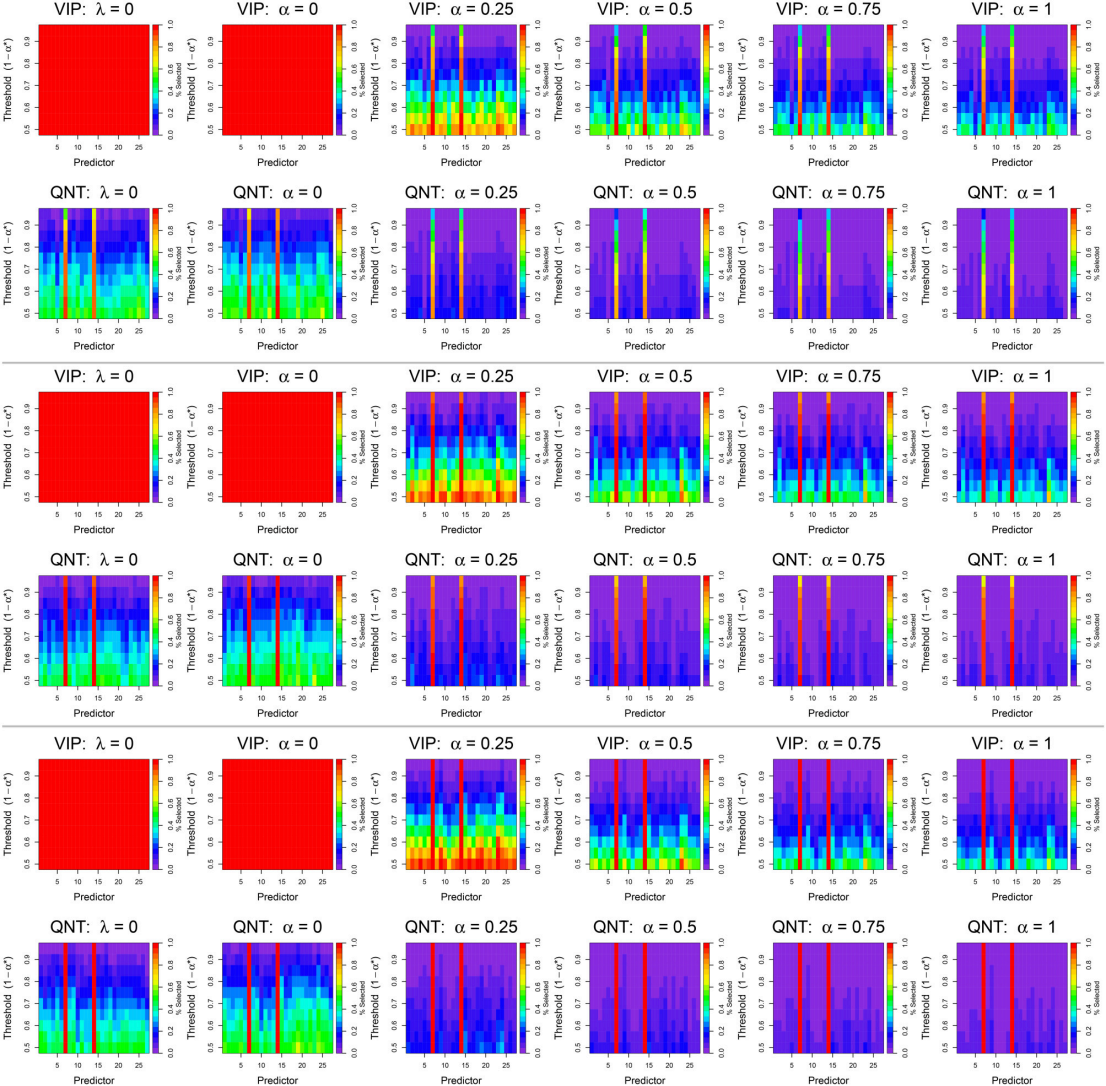
**Results from simulated data analyses with ***n*** = 122 subjects using the VIP approach (top) and the QNT approach (bottom)**. The top two rows display results for a signal-to-noise ratio (SNR) of 1, the middle two rows for a SNR of 0.7, and the bottom two rows for a SNR of 0.5. Each column represents a different regression model: OLS (λ = 0), ridge regression (α = 0), elastic net (α = 0.25, 0.5, 0.75), and the lasso (α = 1). Colored bars indicate how often a specific predictor, i.e., network derived using ICA, was selected at a given significance threshold; the color scale represents the percentage of times that a predictor was selected across 100 simulation replications.

We first present the variable selection results for *n* = 122 (see Figure 4, top), given that this simulation cell (*n* = 122, σ = 1) is most comparable to our real data. Figure 4 can be interpreted in a similar fashion to Figure 3: for each σ level, each subplot displays the variable selection results for each predictor (abscissa) at each threshold (ordinate). The novel aspect of Figure 4 is that colors are used to illustrate the proportion of times that a predictor was deemed significant at a given threshold, where purple indicates a 0% selection rate across simulation replications, and red indicates a 100% selection rate across simulation replications. Consequently, the results in Figure 4 elucidate the Type I (False Positive) and Type II (False Negative) error rates for the different selection rules. In this case a Type I error entails the selection of a non-active predictor (i.e., not ICN 7 or 14) at a given threshold, which is depicted as a cell having a non-purple fill.

Ignoring the VIP-OLS and VIP-ridge results, it is apparent that the active predictors (ICNs 7 and 14) are selected most frequently by both the VIP and QNT approaches, regardless of the chosen tuning parameters. Furthermore, for both the VIP and QNT approaches, we see the selection probabilities decrease as the α tuning parameter increases toward the lasso solution. Again, this result is expected, because the lasso is known to produce sparser models than ridge regression. However, it is interesting to note that, for these data, we found little difference between the CV-MSE for different choices of the α tuning parameter. For most examined sample sizes, the median CV-MSE was nearly identical for all choices of α (see Figure S2); the only noteworthy difference is that the ridge solution produced a noticeably larger CV-MSE with only *n* = 30 subjects.

#### 6.3.2. OLS vs. penalized regression results

Our first overarching goal was to compare OLS vs. penalized regression. To address this aim, we focus on the QNT results, given that the VIP results are uninformative for OLS and ridge. We found that when using OLS regression, the active predictors (ICNs 7 and 14) were selected most frequently across the different thresholds (see Figure 4). At small thresholds (e.g., using confidence intervals with 50% theoretical coverage rates), the OLS and ridge results had Type I Error rates of about 0.6 (indicated by yellow and green cells in the bottom rows), whereas the other QNT results had Type I Error rates of 0.3 or less (indicated by dark blue cells in the bottom rows). Even using the most stringent threshold of 95%, the OLS and ridge results had Type I Error rates exceeding 0.1 (indicated by blue and dark purple cells in the top rows). In contrast, when using a 95% threshold with QNT selection, the non-ridge elastic net results had Type I Error rates less than the nominal 0.05 level (indicated by purple cells in the top rows). These results reveal that OLS and ridge tended to produce more False Positives than did the other elastic net methods.

We also examined model fit for OLS vs. penalized regression using the simulated data (see Figures S2, S3). The boxplots in Figure S3 display the 100 *R*^2^-values (from 100 simulation replications) that were obtained by applying the bias-correction procedure (see Section 4.4) to the variables selected at a 95% QNT threshold. For comparison, we also plot the *R*^2^ results obtained by applying the non-BE OLS solution (OLS-All). Within each subplot, the dashed line displays the true *R*^2^-value treating the *n* = 122 subjects as the population of interest. Note that Figure S3 illustrates the over-fitting tendency of the non-BE OLS solution. In particular, the OLS-All solution tended to over-estimate the true *R*^2^, particularly at smaller sample sizes such as *n* = 30. Interestingly, Figure S3 reveals that ridge regression may be useful for obtaining accurate *R*^2^ estimates in small samples; whereas, the other BE approaches tend to underestimate *R*^2^ in small sample sizes. However, the ridge solution has larger CV-MSE values for smaller samples, see Figure S2.

#### 6.3.3. VIP vs. QNT selection

We next compare the VIP and QNT selection results that are summarized in Figure 4. As a first point, note that in the OLS and ridge solutions, the VIP selected all predictors at all thresholds. As previously mentioned, the VIP can only be meaningfully applied in elastic net situations where α > 0, so this result is not surprising. In contrast, the QNT approach produced relatively consistent results across the six regression methods. Moreover, the VIP approach selected larger models than QNT in every simulation condition (again, this was expected given Theorem 1). Note that with *n* = 122 subjects and a VIP selection threshold of 50%, using the VIP for predictor selection resulted in Type I Error rates that ranged from 0.2 to 1.0 (indicated by red, orange, yellow, green, and blue cells in the bottom rows), regardless of the model signal-to noise ratios (i.e., error standard deviations). As expected, we observed larger Type I Error rates as the elastic tuning parameter α approached zero. Increasing the VIP threshold reduced the Type I Error rates substantially. However, even with α = 1 and a relatively stringent threshold of 70–80%, VIP selection produced False Positive results for many of the inactive predictors.

In contrast to the VIP approach, Figure 4 reveals that the QNT approach produced smaller Type I Error rates. For the non-ridge elastic net solutions (α > 0) with *n* = 122 subjects, the largest observed Type I Error rate (across all predictors and thresholds) was 0.3 when using the QNT. Furthermore, as both the elastic net tuning parameter α and the significance threshold 1 − α^*^ were increased, the Type I Error rate decreased for the inactive predictors. However, this decrease in Type I Error rate came at the cost of a decrease in power (i.e., increase in Type II Error rate). This is evident from Figure 4, given that the QNT approach tended to have slightly lower True Positive rates for the two active coefficients (compared to VIP). However, as Figure 4 reveals, when the signal-to-noise ratio increased (i.e., as σ decreased), QNT had (i) similar power as the VIP and (ii) substantially smaller Type I Error rates.

#### 6.3.4. Sample size and power

To illustrate the influence of sample size on our Type I and Type II Error rates, we plot the variable selection results for the *n* = 90, 60, 30 in Figures S4–S6, respectively. Comparing Figure 4 and Figures S4–S6, it is apparent that reducing the sample size (i) slightly decreased our Type I error rates, and (ii) severely increased our Type II error rates. With the realistic error standard deviation of σ = 1 and data from only *n* = 60 subjects, our power to detect true predictors was 0.11 or less for both VIP and QNT using the stringent threshold of 95% (indicated by blue cells for the active predictors in the top rows). Consequently, for smaller samples, researchers may need to set 1 − α^*^ larger. However, Figures S4–S6 reveal that as σ decreases, both the VIP and QNT selection results improve substantially (indicated by brighter colored cells for the active predictors in the top rows). Finally, comparing VIP vs. QNT selection, we see that VIP selection results in larger Type I error rates and smaller Type II error rates for the same selection threshold; this finding was expected, given the result in Theorem 1.

## 7. Conclusions

### 7.1. Summary of findings

As psychology and neuroscience research continue to evolve, multivariate methods for analyzing high-dimensional data are becoming more accessible. Methods such as ICA enable researchers to characterize behavioral mechanisms across the whole brain by decomposing the neural signal from millions of voxels into a smaller number of components with interpretable functions (Beckmann, 2012; Duff et al., 2012). However, the use of traditional regression approaches in this context is problematic when the number of networks begins to approach the number of subjects. Even with adequate statistical power, estimated OLS coefficients from traditional regression are unstable when substantial inter-predictor collinearity is present. Issues of high collinearity are especially concerning for fMRI connectivity analyses (Sporns, 2013) where, due to the high cost of data collection, sample sizes are frequently modest and predictors are highly correlated. In this article, we describe three penalized regression models—ridge regression, the elastic net, and the lasso—that are particularly well suited for the analysis of fMRI data as these methods do not suffer from the aforementioned limitations of OLS regression.

Our first overarching goal in this paper was to compare the performance of penalized regression methods with OLS regression in the context of correlated fMRI data. To accomplish this goal, we demonstrated procedures using both real and simulated neuroimaging data. We found that both the elastic net and lasso selected two of the three networks containing the greatest number of insula voxels. The OLS and ridge regression models retained the most predictors, whereas the elastic net and lasso models retained the fewest predictors. Moreover, our simulation results indicated that the elastic net and lasso had lower False Positive Rates (i.e., Type I Errors) when compared with OLS and ridge regression. Surprisingly, we did not observe differences in CV-MSE across the elastic net variations. Although the three penalized regression techniques produced comparable results in our example, this high degree of method comparability is not guaranteed for all neuroimaging data. A Monte Carlo procedure such as that suggested in Section 6 can be applied to determine which penalized regression model performs under different data conditions.

Our second overarching goal was to compare the performance of our proposed bootstrapped QNT confidence interval approach to Bunea et al.'s (2011) VIP approach. To accomplish this goal, we presented new theoretical results connecting the VIP and QNT selection rules, and we thoroughly compared the approaches using both real and simulated neuroimaging data. With respect to our real data findings, the QNT approach produced sparse results (i.e., few selected predictors) across all models. Additionally, from a practical standpoint, we found it problematic to detect an optimal VIP threshold as many predictors were retained at 70–90% cutoff values. Our simulation study similarly indicated that the QNT approach yielded more conservative results than the VIP, as indicated by lower False Positive rates. This result is especially salient in functional neuroimaging research, which is an area that may be highly susceptible to False Positives (Carp, 2012).

Finally, the real data analyses reveal that insula network coherence predicts individual differences in externalizing tendencies. These findings supplement past research, which has found that insula network integrity (e.g., anterior insula-anterior cingulate cortex) may underlie a range of psychiatric disorders (see Naqvi and Bechara, 2009; Wisner et al., 2013b; Carroll et al., 2015). For instance, a recent meta-analysis (using the revised activation likelihood estimation algorithm) found reduced gray matter loss in the anterior insula and dorsal anterior cingulate across six diagnostic groups, e.g., schizophrenia, addiction, and anxiety (Goodkind et al., 2015). Our findings therefore support research that has linked insula function and morphology with impulse-related disorders, such as addiction. Moreover, because we utilized a community control sample, we highlight the presence of insula-externalizing relationships even in the absence of a clinical diagnosis.

### 7.2. Alternative applications

In coming years, multivariate data-driven techniques for data reduction, model generation, and cross-validation will become increasingly valuable as researchers continue to investigate clinically meaningful differences in large-scale brain networks. As such, the methods demonstrated here apply to other types of neuroimaging studies, beyond our investigation of individual differences in ICA-derived networks. For instance, penalized regression may be valuable for analyzing output obtained from a graph-theoretical decomposition, where researchers face similar challenges of modeling numerous brain-derived metrics, e.g., hundreds of between network variables (Bullmore and Sporns, 2009). Comparable situations can also arise in multi-seed connectivity analyses, particularly if a researcher is interested in pairwise connections between vast numbers of brain regions (see Camchong et al., 2011). This alternative application is particularly salient given that between-network connectivity is pertinent for evaluating brain health, such as age-related changes in functional connectivity and psychopathology (Baker et al., 2014; Grady et al., 2016).

Furthermore, the applicability of penalized regression in neuroimaging research is not restricted to individual differences analyses. For example, similar couplings of multivariate methods are useful for researchers examining within-subject variables, such as the relation between ICA-derived networks and behavioral responses or task-related hemodynamic models (Calhoun et al., 2012). Researchers may also be interested in characterizing group differences via temporal or spatial network information (Jafri et al., 2008; Ma et al., 2014); this latter aim has become a recent focus in the schizophrenia and bipolar research literatures (Calhoun and Adali, 2012; Calhoun et al., 2014).

Lastly, we want to emphasize that penalized regression can also be utilized when the variables of interest are voxels, as opposed to brain networks or regions of interest; however, we remind the reader of the motivation to use a set of functionally homogeneous predictors (see Section 5.1). Note that the use of voxels as predictors could pose a problem for the lasso, which tends to arbitrarily select one variable (voxel) out of a group of correlated variables (voxels), see Section 4.2. For voxel-wise analyses, we recommend applying either the elastic net (Zou and Hastie, 2005) or the group lasso (Yuan and Lin, 2006) to encourage functionally homogeneous voxels to be either selected or excluded from the model as a group.

### 7.3. Limitations

We now mention the limitations of our proposed QNT approach. Our simulation results indicate that the QNT method was slightly less powerful than the VIP method for the same significance threshold. More specifically, the VIP demonstrated a higher hit rate for our two active predictors as sample size decreased, but with a concomitant higher False Positive rate. This effect was more pronounced for smaller sample sizes, e.g., *n* ≤ 60 subjects. Thus, for typical SNRs encountered in behavioral neuroimaging research, a sample size of approximately *n* = 100 may be required. However, ridge regression may prove useful for smaller samples (cf., Figure 4 and Figures S2–S6).

### 7.4. General recommendations

When applying penalized regression, a neuroscientist often wants to know which penalized regression method will be most useful for their particular data. The methodology presented in this paper can help answer that question. To compare the performance of different penalized regression approaches on one's own data, we recommend an approach similar to that employed in Section 5. In particular, the bootstrap QNT approach can be applied to produce a graphic similar to Figure 3, which can be useful for assessing the sensitivity of the bootstrap enhanced penalized regression to the λ and α tuning parameters (i.e., for assessing how λ and α affect variable selection). Furthermore, to assess the effectiveness of the penalized regression for one's own data, it is possible to use a Monte Carlo approach similar to that used in Section 6. Specifically, a Monte Carlo simulation can be used to assess the performance (e.g., Type I and Type II Error rates) of the methods under different situations, e.g., sample sizes, signal-to-noise ratios, sparsity levels, correlation structures, etc. The R code that we provide in the Supplementary Material can be easily modified for such analyses.

### 7.5. Concluding remarks

The utility and power of penalized regression in the clinical neuroscience field will only amplify as increasing numbers of large fMRI datasets become publicly available. The Human Connectome Project (Van Essen et al., 2012) is one such example, where researchers have access to an ever-expanding fMRI dataset (end of goal of *n* = 1200). This advance in the neuroimaging field stresses the need to adopt newer statistical techniques, like penalized regression, to accommodate high dimensional neuroimaging data. In this article, we have provided both theoretical and Monte Carlo results to advance the use of penalized regression with neuroimaging data. In an online supplement, we also provide open-source R code to select penalized regression tuning parameters and to evaluate regression coefficient significance using nonparametric bootstrap methods. We hope that these resources will help other researchers to better understand their functional neuroimaging data.

## Author contributions

SA, NH, and NW contributed to the design, analysis, and writing of the manuscript. CM contributed to the writing and editing of the manuscript. CD contributed his data and edited the final manuscript. AM contributed to the editing of the manuscript.

### Conflict of interest statement

The authors declare that the research was conducted in the absence of any commercial or financial relationships that could be construed as a potential conflict of interest.

## Footnotes

^1^We also note that the discussed penalized regression methods can be equivalently applied to other subtypes of neuroimaging data, such as voxel-wise activity or between-network connectivity metrics. However, a voxel-wise approach may not be optimal, as, ideally, one would like each of the variables in the pool to have some meaningful functional distinction. Voxels do not meet that criterion, whereas components do because they reflect networks that map onto cognitive/emotional processes and behaviors, e.g., Laird et al. (2011). Thus, from a theoretical perspective, it is valuable to first reduce neuroimaging data to functionally homogeneous components before examining brain-behavior associations. We refer the reader to Section 7.2 for a discussion of alternative applications of the proposed penalized regression approach.

^2^We used the following scanning parameters: gradient-echo echo-planar imaging of 150 volumes; repetition time (TR) = 2 s; echo time (TE) = 28 ms; flip angle = 80°; voxel size = 3.5 × 3.5 × 3.5 mm.

^3^http://fsl.fmrib.ox.ac.uk/fsl/fslwiki/MELODIC

^4^Although our specific example focuses on how individuals differ in terms of functionality within group-defined networks, researchers may alternatively explore relations between individual differences in network topography and external behaviors/traits via penalized regression. Single-subject ICA has the benefit of accommodating unique spatial and temporal features, but has disadvantages in that the resulting components can be noisy and are not necessarily unmixed equivalently for every subject (Erhardt et al., 2011). Consequently, in this paper we use the group-defined networks to ensure comparability across subjects.
