# Supplementary material for: Bootstrap Enhanced Penalized Regression for Variable Selection with Neuroimaging Data
Source: Front Neurosci. 2016 Jul 28;10:344. doi: 10.3389/fnins.2016.00344 (PMC4964314; doi:10.3389/fnins.2016.00344)
Supplement: Supplementary file 1 [file DataSheet1.pdf]

Supplementary Online Material: Bootstrap Enhanced Penalized Regression for Variable Selection with Neuroimaging Data

Samantha V. Abram, Nathaniel E. Helwig, Craig A. Moodie, Colin G. DeYoung, Angus W. MacDonald III, and Niels G. Waller

*Correspondence:
Niels G. Waller
nwaller@umn.edu

1 MULTIVARIATE FUNCTIONAL CONNECTIVITY ANALYSIS

Multivariate methods for the analysis of fMRI data have become a popular alternative to univariate or bivariate techniques as they are designed to yield statistically optimal summaries of large quantities of data (Cox and Savoy, 2003). Multivariate approaches are particularly pertinent to functional connectivity studies that aim to characterize the communication of regions across the brain (Van De Ven et al., 2004). Historically, functional connectivity studies relied on seed based methods, where the average time series of one voxel cluster (the seed) was correlated with the time series from voxel clusters in other brain regions (Biswal et al., 1995; Lowe et al., 1998). Previous literature has identified at least two potential biases associated with seed-based techniques.

First, seed based techniques are biased by the strategies used to select the seed region and its theorized correlates. The brain does not contain a single hub with projections throughout the cortex. Instead, the brain is composed of numerous spatially distributed and functionally related regions (or networks) that each have multiple hubs (Bullmore and Sporns, 2012; Tomasi and Volkow, 2011; Van den Heuvel and Pol, 2010). Second, different types of fMRI noise, such as variance associated with physiological artifacts (e.g., head motion, cardiac function), can introduce bias into subsequent analyses (Beckmann et al., 2005; Liu et al., 2012; Van De Ven et al., 2004). A simple bivariate statistic, such as a correlation, cannot account for variance associated with physiological artifacts (Beckmann et al., 2005). Multivariate methods are more suitable in this context as they potentially control for such variables.

An increasingly used multivariate alternative to traditional univariate approaches is the application of ICA to fMRI data (Beckmann and Smith, 2004; Jafri et al., 2008). Briefly, ICA is a "temporal model-free" method that uses blind source separation to identify the underlying multivariate signals and structured noise in the data (De Luca et al., 2006; Smith et al., 2004). ICA accomplishes this decomposition without the use of a temporal model (Beckmann and Smith, 2004; Beckmann et al., 2005) or assumptions about the underlying biology (Esposito et al., 2005; Ramnani et al., 2004). Thus, ICA is a powerful data-driven approach for evaluating natural functional connectivity (Calhoun et al., 2009).

In the context of fMRI, ICA decomposes 4-dimensional data (3 spatial, 1 time) into spatial maps and their corresponding time courses. Components derived from fMRI data have been referred to as intrinsic connectivity networks (ICNs) as they are believed to reflect the brain's inherent architecture both at rest and during task completion (Laird et al., 2011; Smith et al., 2009). One strength of this methodology is its capacity to reduce signals from millions of voxels into a set of functionally interpretable components

(Duff et al., 2012). However, many researchers are interested in how the strengths (or weaknesses) of ICNs underlie psychopathology (Bressler and Menon, 2010; Calhoun and Adali, 2012).

The ICA process itself cannot tell us which networks are important to one or more criteria of interest. For instance, researchers may be interested in whether spatial or temporal characteristics of the independent networks predict self-report measures (Wisner et al., 2013b), task-related BOLD activation (Mennes et al., 2010; Moodie et al., 2014), or group differences (Calhoun et al., 2012). Thus, additional methods are needed to identify the subset of substantively important networks. Ordinary least squares (OLS) regression models have often been used for this purpose (Choi et al., 2013; Eaton et al., 2012; Kim et al., 2009; Mennes et al., 2010; Stevens et al., 2007); however, as we explain in the manuscript, OLS regression may be ill-suited for this task.

2 THE NEURAL BASIS OF EXTERNALIZING

The externalizing spectrum includes a range of traits and behaviors, such as impulsivity, aggression, and substance use (Krueger et al., 2007). These traits have been shown to occur along a continuum from clinical to non-clinical populations. Moreover, externalizing problems can produce substantial psychological and functional ramifications, which in turn, engender significant financial costs to the community (Woltering et al., 2011). As such, researchers seek to elucidate the neural mechanisms that underlie externalizing behaviors and related psychopathology. A breadth of literature has linked the prefrontal cortex and ventral striatum with externalizing (Crews and Boettiger, 2009). More recently, several researchers have demonstrated the importance of the insula in externalizing psychopathology (Carroll et al., 2015; Goldstein et al., 2009; Naqvi and Bechara, 2009; Wisner et al., 2013b; Xue et al., 2010). This burgeoning research linking the insula with externalizing serves as the basis for our analyses. In this paper, we present findings that relate insula connectivity with externalizing traits in a non-clinical sample.

3 APPLYING ICA TO RESTING FMRI DATA

The full sample of data includes an additional 122 female subjects that were not included in the penalized regression analyses presented in the paper.[1] ICNs were generated using a meta-ICA procedure that was designed to optimize network reliability using the full sample of 244 participants (Moodie et al., 2014; Poppe et al., 2013; Wisner et al., 2013a). This procedure entailed running 25 temporal concatenation (model-free and multi-subject) group-level probabilistic ICAs. Each lower-level ICA included a random subject order of 80 participants as inputs (sampled from the 244), and was constrained to a dimensionality of 60, due to computational/hardware limitations and to reduce chances of over-fitting. The resulting 60 components from each lower-level ICA were then concatenated into a single file ($60 \times 25 = 1500$ components total). The concatenated file was then employed as the input to a single meta-level MELODIC (hence, meta-ICA) to derive the 60 most consistent group-level components. Our choices to include 25 random subject orders with a dimensionality constraint of 60 were based on reliability analyses detailed by Poppe et al. (2013).

4 SUPPLEMENTARY FIGURES

[1] We included only males in our demonstration given notable sex differences in externalizing psychopathology (Eaton et al., 2012; Kramer et al., 2008; NolenHoeksema, 2004) and insula morphology and function (Lee et al., 2009; Moriguchi et al., 2014; Stevens and Hamann, 2012; Tanabe et al., 2013).

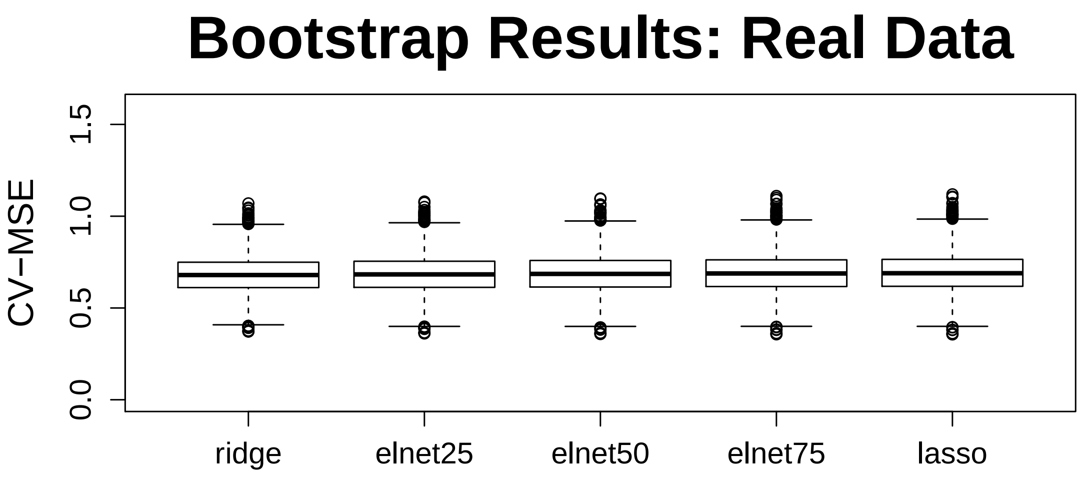

Figure S1. Visualization of bootstrapped model fit (cross-validated mean-squared error, or CV-MSE) for real data across penalized regression methods.

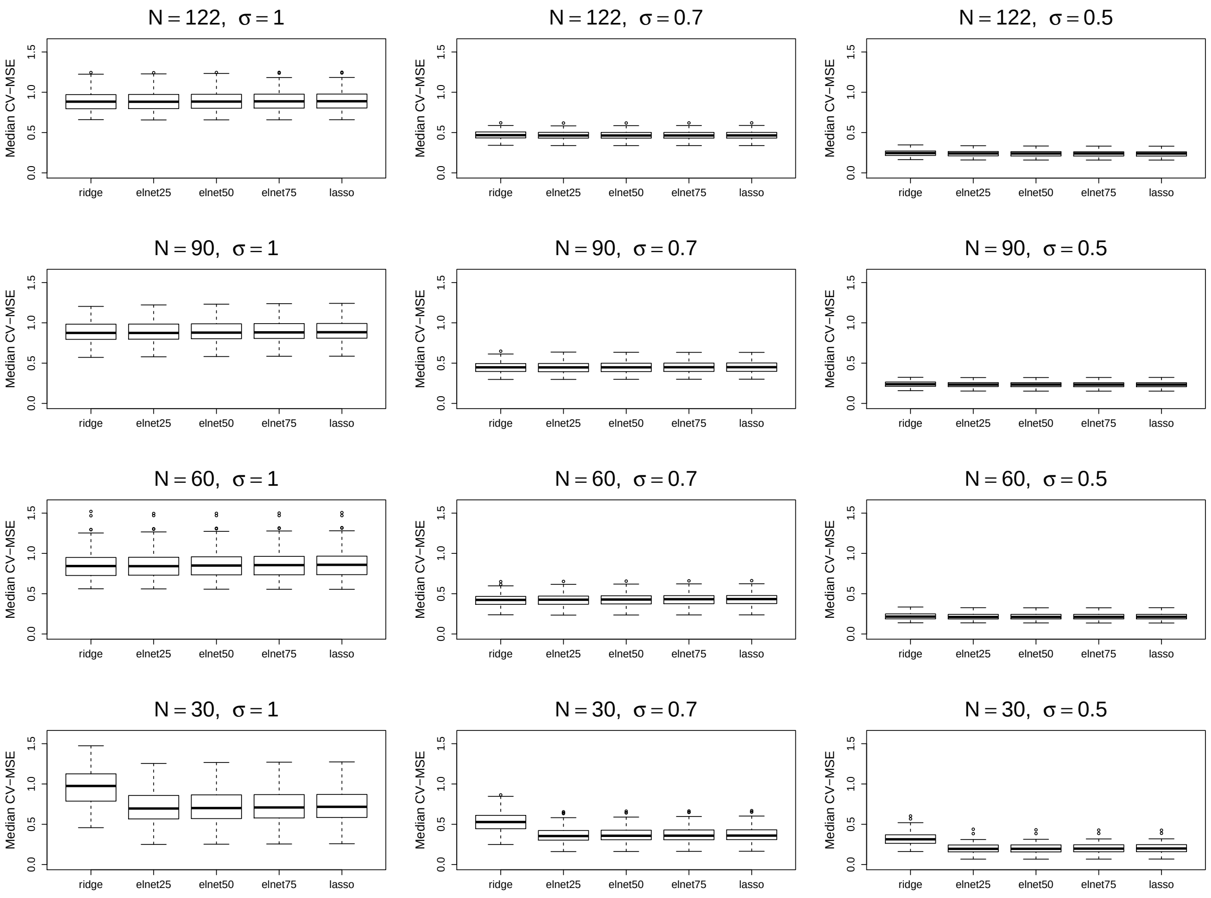

Figure S2. Illustrations of model fit (cross-validated mean-squared error or CV-MSE) across different sample sizes and signal-to-noise ratios for OLS regression when the model includes all possible predictors (ols-all), OLS regression (ols), ridge regression (ridge), elastic net where $\alpha = 0.25$ (elnet25), elastic net where $\alpha = 0.5$ (elnet50), elastic net where $\alpha = 0.75$ (elnet75), and the lasso (lasso).

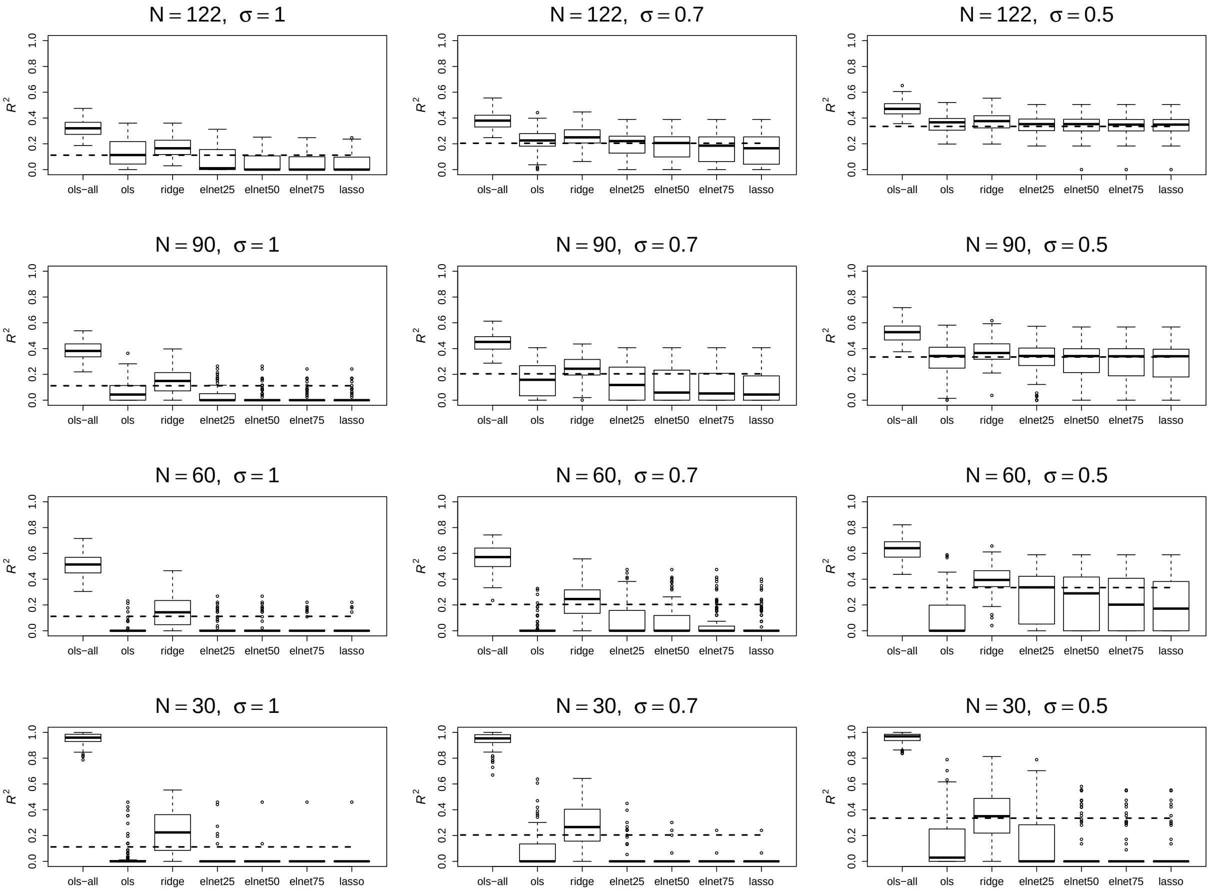

Figure S3. Illustrations of model fit (R^2) across different sample sizes and signal-to-noise ratios for OLS regression when the model includes all possible predictors (ols-all), OLS regression (ols), ridge regression (ridge), elastic net where $\alpha = 0.25$ (elnet25), elastic net where $\alpha = 0.5$ (elnet50), elastic net where $\alpha = 0.75$ (elnet75), and the lasso (lasso).

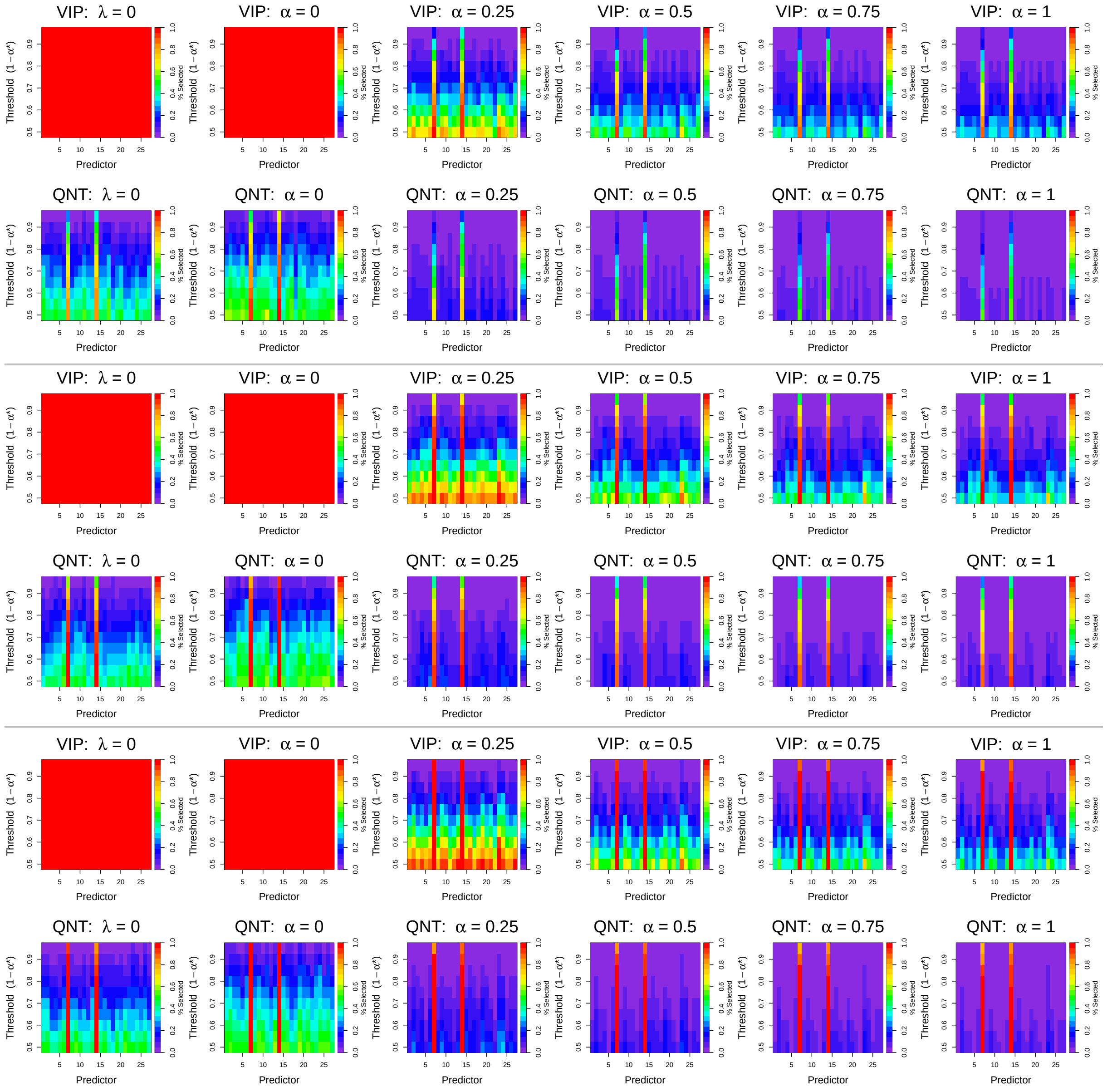

Figure S4. Results from simulated data analyses with $n = 90$ subjects using the VIP approach (top) and the QNT approach (bottom). The top two rows display results for a signal-to-noise ratio (SNR) of 1, the middle two rows for a SNR of 0.7, and the bottom two rows for a SNR of 0.5. Each column represents a different regression model: OLS ($\lambda = 0$), ridge regression ($\alpha = 0$), elastic net ($\alpha = 0.25, 0.5, 0.75$), and the lasso ($\alpha = 1$). Colored bars indicate how often a specific predictor, i.e., network derived using ICA, was selected at a given significance threshold; the color scale represents the percentage of times that a predictor was selected across 100 simulation replications.

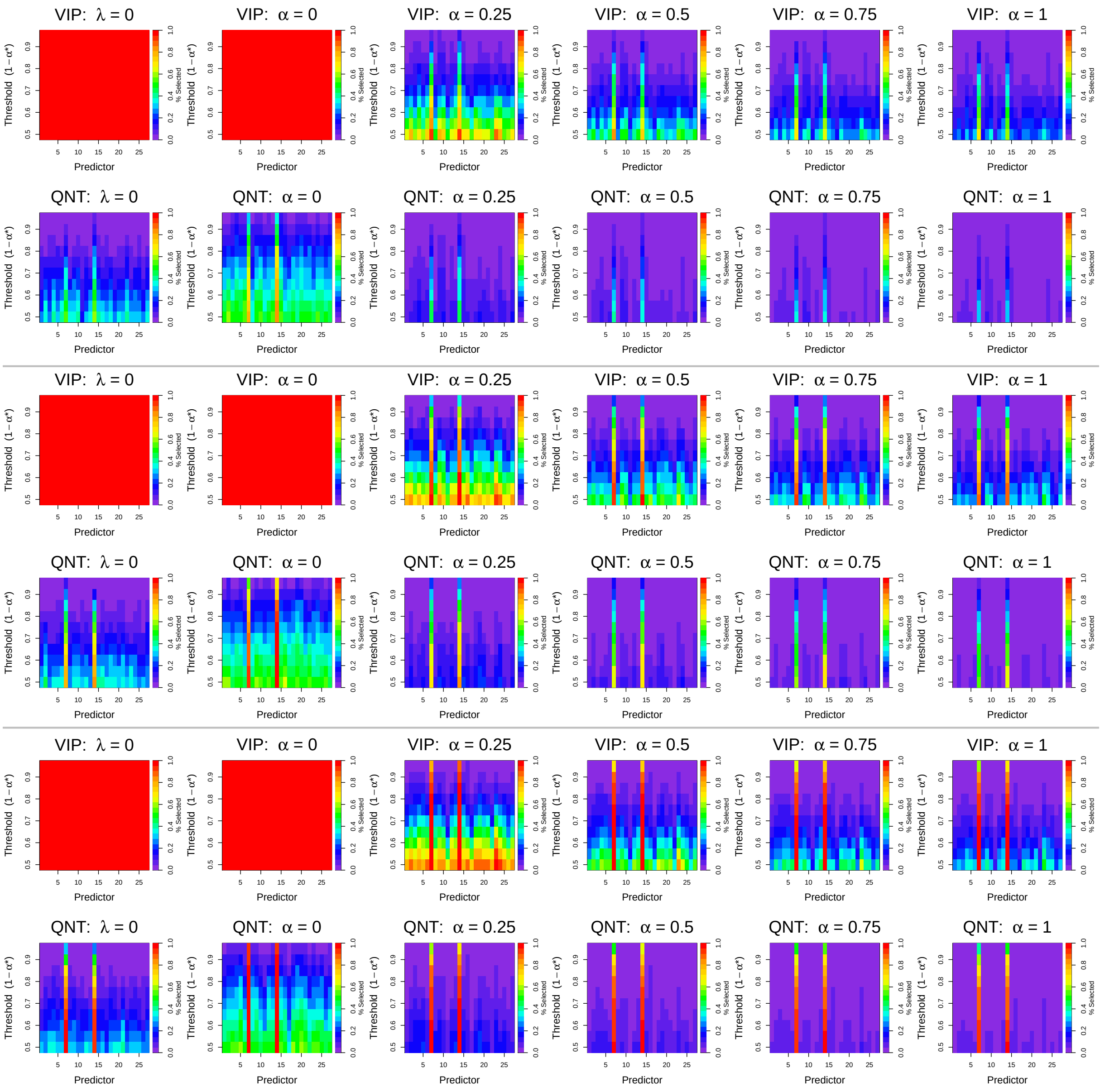

Figure S5. Results from simulated data analyses with $n = 60$ subjects using the VIP approach (top) and the QNT approach (bottom). The top two rows display results for a signal-to-noise ratio (SNR) of 1, the middle two rows for a SNR of 0.7, and the bottom two rows for a SNR of 0.5. Each column represents a different regression model: OLS ($\lambda = 0$), ridge regression ($\alpha = 0$), elastic net ($\alpha = 0.25, 0.5, 0.75$), and the lasso ($\alpha = 1$). Colored bars indicate how often a specific predictor, i.e., network derived using ICA, was selected at a given significance threshold; the color scale represents the percentage of times that a predictor was selected across 100 simulation replications.

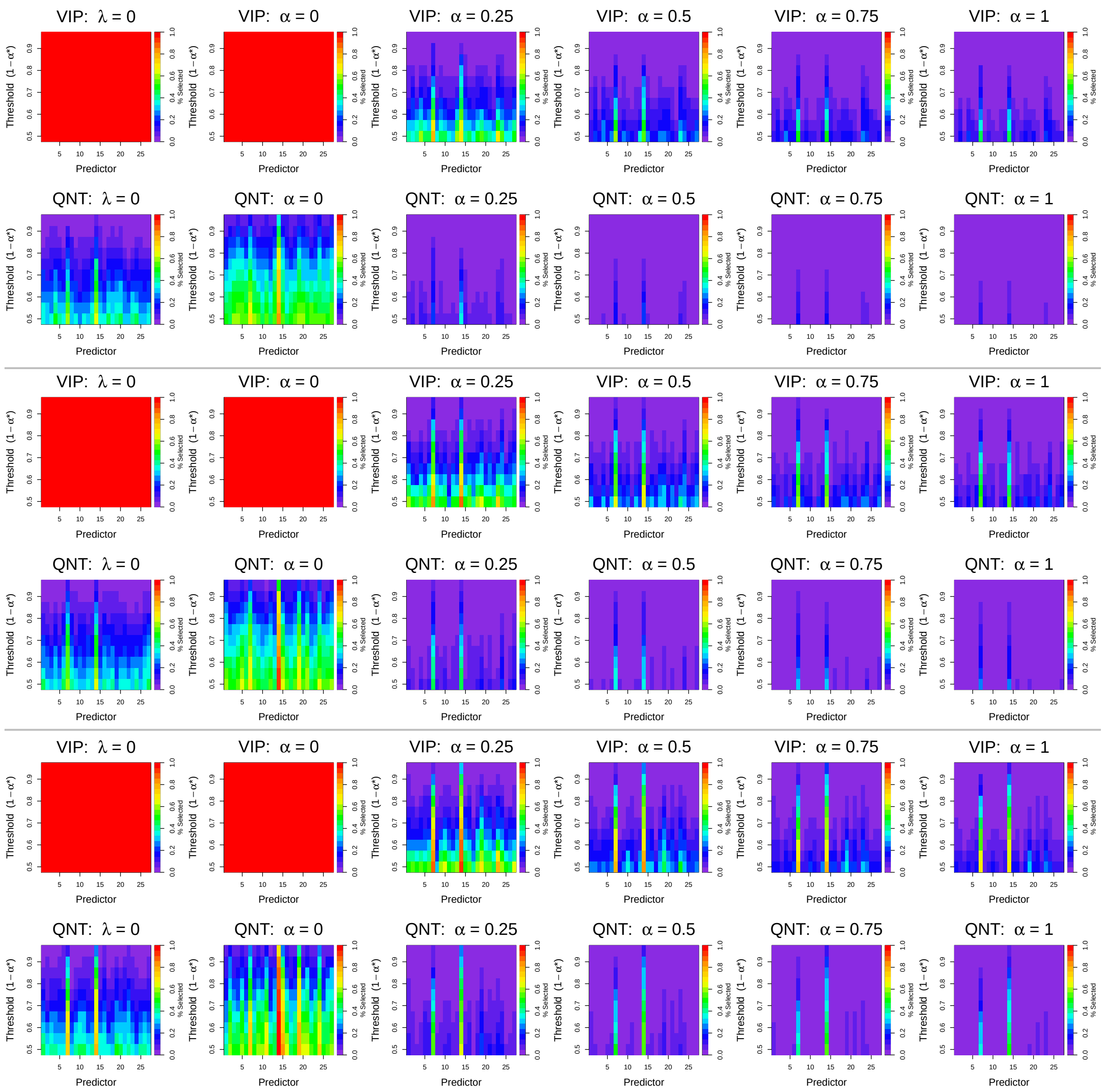

Figure S6. Results from simulated data analyses with $n = 30$ subjects using the VIP approach (top) and the QNT approach (bottom). The top two rows display results for a signal-to-noise ratio (SNR) of 1, the middle two rows for a SNR of 0.7, and the bottom two rows for a SNR of 0.5. Each column represents a different regression model: OLS ($\lambda = 0$), ridge regression ($\alpha = 0$), elastic net ($\alpha = 0.25, 0.5, 0.75$), and the lasso ($\alpha = 1$). Colored bars indicate how often a specific predictor, i.e., network derived using ICA, was selected at a given significance threshold; the color scale represents the percentage of times that a predictor was selected across 100 simulation replications.

REFERENCES

Beckmann, C. F., DeLuca, M., Devlin, J., and Smith, S. (2005). Investigations into resting-state connectivity using independent component analysis. *Philosophical Transactions of the Royal Society B: Biological Sciences* 360, 1001–1013

Beckmann, C. F. and Smith, S. M. (2004). Probabilistic independent component analysis for functional magnetic resonance imaging. *IEEE Transactions on Medical Imaging* 23, 137–152

Biswal, B., Yetkin, F. Z., Haughton, V. M., and Hyde, J. S. (1995). Functional connectivity in the motor cortex of resting human brain using echo-planar mri. *Magnetic Resonance in Medicine* 34, 537–541

Bressler, S. and Menon, V. (2010). Large-scale brain networks in cognition: emerging methods and principles. *Trends in Cognitive Sciences* 14, 277–290

Bullmore, E. and Sporns, O. (2012). The economy of brain network organization. *Nature Reviews Neuroscience* 13, 336–349

Calhoun, V. and Adali, T. (2012). Multisubject independent component analysis of fmri: A decade of intrinsic networks, default mode, and neurodiagnostic discovery. *IEEE Reviews in Biomedical Engineering* 5, 60–73

Calhoun, V., Liu, J., and Adali, T. (2009). A review of group ica for fmri data and ica for joint inference of imaging, genetic, and erp data. *NeuroImage* 45, S163–S172

Calhoun, V., Sui, J., Kiehl, K., Turner, J., Allen, E., and Pearlson, G. (2012). Exploring the psychosis functional connectome: Aberrant intrinsic networks in schizophrenia and bipolar disorder. *Frontiers in Psychiatry* 2:75, 1–13

Carroll, A., Sutherland, M., Salmeron, B., Ross, T., and Stein, E. (2015). Greater externalizing personality traits predict less error-related insula and anterior cingulate cortex activity in acutely abstinent cigarette smokers. *Addiction Biology* 20, 377–389

Choi, J., Jeong, B., Lee, S., and Go, H. (2013). Aberrant development of functional connectivity among resting state-related functional networks in medication-naive ADHD children. *PLoS One* 8, e83516

Cox, D. and Savoy, R. (2003). Functional magnetic resonance imaging (fmri) “brain reading”: Detecting and classifying distributed patterns of fmri activity in human visual cortex. *NeuroImage* 19, 261–270

Crews, F. and Boettiger, C. (2009). Impulsivity, frontal lobes and risk for addiction. *Pharmacology Biochemistry and Behavior* 93, 237–247

De Luca, M., Beckmann, C. F., De Stefano, N., Matthews, P. M., and Smith, S. M. (2006). fMRI resting state networks define distinct modes of long-distance interactions in the human brain. *Neuroimage* 29, 1359–1367

Duff, E. P., Trachtenberg, A. J., Mackay, C. E., Howard, M. A., Wilson, F., Smith, S. M., et al. (2012). Task-driven ICA feature generation for accurate and interpretable prediction using fMRI. *Neuroimage* 60, 189–203

Eaton, N., Keyes, K., Krueger, R., Balsis, S., Skodol, A., Markonand B.F. Grant, K., et al. (2012). An invariant dimensional liability model of gender differences in mental disorder prevalence: evidence from a national sample. *Journal of Abnormal Psychology* 121, 282–288

Esposito, F., Scarabino, T., Hyvärinen, A., Himberg, J., Formisano, E., Comani, S., et al. (2005). Independent component analysis of fmri group studies by self-organizing clustering. *NeuroImage* 25, 193–205

Goldstein, R., Craig, A., Bechara, A., Garavan, H., Childress, A., Paulus, M., et al. (2009). The neurocircuitry of impaired insight in drug addiction. *Trends in Cognitive Sciences* 13, 372–380

Jafri, M. J., Pearlson, G. D., Stevens, M., and Calhoun, V. D. (2008). A method for functional network connectivity among spatially independent resting-state components in schizophrenia. *Neuroimage* 39, 1666–1681

Kim, D., Manoach, D., Mathalon, D., Turner, J., Mannell, M., Brown, G., et al. (2009). Dysregulation of working memory and default-mode networks in schizophrenia using independent component analysis, an fbirn and mcic study. *Human Brain Mapping* 30, 3795–3811

Kramer, M., Krueger, R., and Hicks, B. (2008). The role of internalizing and externalizing liability factors in accounting for gender differences in the prevalence of common psychopathological syndromes. *Psychological Medicine* 38, 51–61

Krueger, R., Markon, K., Patrick, C., Benning, S., and Kramer, M. (2007). Linking antisocial behavior, substance use, and personality: An integrative quantitative model of the adult externalizing spectrum. *Journal of Abnormal Psychology* 116, 645–666

Laird, A., Fox, P., Eickhoff, S., Turner, J., Ray, K., McKay, D., et al. (2011). Behavioral interpretations of intrinsic connectivity networks. *Journal of Cognitive Neuroscience* 23, 4022–4037

Lee, T., Chan, C., Leung, A., Fox, P., and Gao, J. (2009). Sex-related differences in neural activity during risk taking: An fmri study. *Cerebral Cortex* 19, 1303–1312

Liu, X., Zhu, X.-H.-H., Qiu, P., and Chen, W. (2012). A correlation-matrix-based hierarchical clustering method for functional connectivity analysis. *Journal of Neuroscience Methods* 211, 94–102

Lowe, M., Mock, B., and Sorenson, J. (1998). Functional connectivity in single and multislice echoplanar imaging using resting-state fluctuations. *NeuroImage* 7, 119–132

Mennes, M., Kelly, C., Zuo, X., Di Martino, A., Biswal, B., Castellanos, F., et al. (2010). Inter-individual differences in resting-state functional connectivity predict task-induced BOLD activity. *NeuroImage* 50, 1690–1701

Moodie, C. A., Wisner, K., and MacDonald III, A. (2014). Characteristics of canonical intrinsic connectivity networks across tasks and monozygotic twin pairs. *Human Brain Mapping* 35, 5532–5549

Moriguchi, Y., Touroutoglou, A., Dickerson, B., and Barrett, L. (2014). Sex differences in the neural correlates of affective experience. *Social Cognitive and Affective Neuroscience* 9, 591–600

Naqvi, N. and Bechara, A. (2009). The hidden island of addiction: the insula. *Trends in Neurosciences* 32, 56–67

NolenHoeksema, S. (2004). Gender differences in risk factors and consequences for alcohol use and problems. *Clinical Psychology Review* 24, 981–1010

Poppe, A., Wisner, K., Atluri, G., Lim, K., Kumar, V., and MacDonald III, A. (2013). Toward a neurometric foundation for probabilistic independent component analysis of fmri data. *Cognitive, Affective, & Behavioral Neuroscience* 13, 641–659

Ramnani, N., Behrens, T., Penny, W., and Matthews, P. (2004). New approaches for exploring anatomical and functional connectivity in the human brain. *Biological Psychiatry* 56, 613–619

Smith, S., Fox, P., Miller, K., Glahn, D., Fox, P., Mackay, C., et al. (2009). Correspondence of the brain's functional architecture during activation and rest. *Proceedings of the National Academy of Sciences* 106, 13040–13045

Smith, S., Jenkinson, M., Woolrich, M., Beckmann, C., Behrens, T., Johansen-Berg, H., et al. (2004). Advances in functional and structural MR image analysis and implementation as FSL. *NeuroImage* 23, S208–S219

Stevens, J. and Hamann, S. (2012). Sex differences in brain activation to emotional stimuli: A meta-analysis of neuroimaging studies. *Neuropsychologia* 50, 1578–1593

Stevens, M., Kiehl, K., Pearlson, G., and Calhoun, V. (2007). Functional neural networks underlying response inhibition in adolescents and adults. *Behavioural Brain Research* 181, 12–22

Tanabe, J., York, P., Krmpotich, T., Miller, D., Dalwani, M., Sakai, J., et al. (2013). Insula and orbitofrontal cortical morphology in substance dependence is modulated by sex. *American Journal of Neuroradiology* 34, 1150–1156

Tomasi, D. and Volkow, N. (2011). Association between functional connectivity hubs and brain networks. *Cerebral Cortex* 21, 2003–2013

Van De Ven, V., Formisano, E., Prvulovic, D., Roeder, C., and Linden, D. (2004). Functional connectivity as revealed by spatial independent component analysis of fmri measurements during rest. *Human Brain Mapping* 22, 165–178

Van den Heuvel, M. P. and Pol, H. E. H. (2010). Exploring the brain network: A review on resting-state fMRI functional connectivity. *European Neuropsychopharmacology* 20, 519–534

Wisner, K., Atluri, G., Lim, K., and MacDonald III, A. (2013a). Neurometrics of intrinsic connectivity networks at rest using fmri: retest reliability and cross-validation using a meta-level method. *NeuroImage* 76, 236–251

Wisner, K., Patzelt, E., Lim, K., and MacDonald III, A. (2013b). An intrinsic connectivity network approach to insula-derived dysfunctions among cocaine users. *American Journal of Drug and Alcohol Abuse* 39, 403–413

Woltering, S., Granic, I., Lamm, C., and Lewis, M. (2011). Neural changes associated with treatment outcome in children with externalizing problems. *Biological Psychiatry* 70, 873–879

Xue, G., Lu, Z., Levin, I., and Bechara, A. (2010). The impact of prior risk experiences on subsequent risky decision-making: the role of the insula. *NeuroImage* 50, 709–716
